# METTL3 from Target Validation to the First Small-Molecule Inhibitors: A Medicinal Chemistry Journey

**DOI:** 10.1021/acs.jmedchem.2c01601

**Published:** 2023-01-24

**Authors:** Francesco Fiorentino, Martina Menna, Dante Rotili, Sergio Valente, Antonello Mai

**Affiliations:** † Department of Drug Chemistry and Technologies, Sapienza University of Rome, Piazzale Aldo Moro 5, 00185 Rome, Italy; ‡ Pasteur Institute, Cenci-Bolognetti Foundation, Sapienza University of Rome, Piazzale Aldo Moro 5, 00185 Rome, Italy

## Abstract

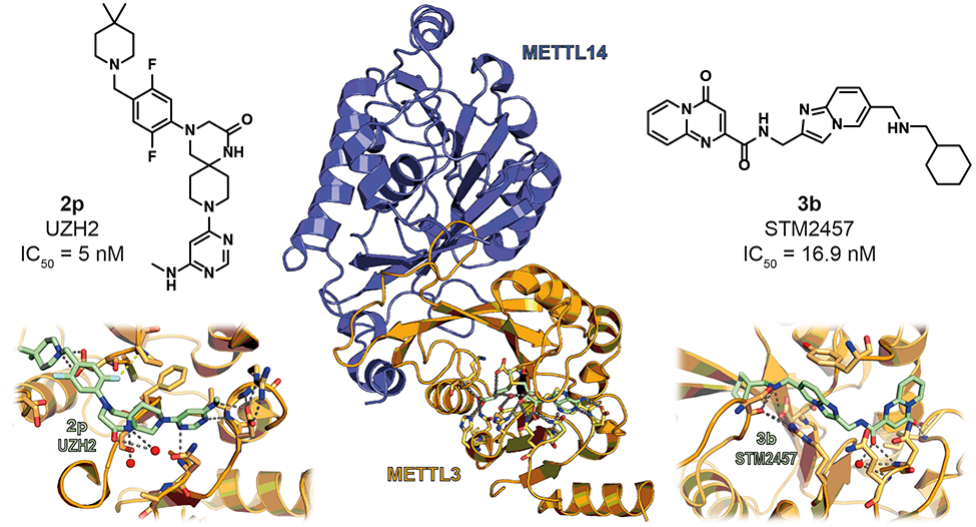

RNA methylation is a critical mechanism for regulating the transcription and translation of specific sequences or for eliminating unnecessary sequences during RNA maturation. METTL3, an RNA methyltransferase that catalyzes the transfer of a methyl group to the *N*^6^-adenosine of RNA, is one of the key mediators of this process. METTL3 dysregulation may result in the emergence of a variety of diseases ranging from cancer to cardiovascular and neurological disorders beyond contributing to viral infections. Hence, the discovery of METTL3 inhibitors may assist in furthering the understanding of the biological roles of this enzyme, in addition to contributing to the development of novel therapeutics. Through this work, we will examine the existing correlations between METTL3 and diseases. We will also analyze the development, mode of action, pharmacology, and structure–activity relationships of the currently known METTL3 inhibitors. They include both nucleoside and non-nucleoside compounds, with the latter comprising both competitive and allosteric inhibitors.

## Introduction

Over the past few years, many studies have focused on RNA modifications. These are involved in the transcription and maturation of RNA, a mechanism that contributes to the maintenance of cellular homeostasis. RNA is known to be critical for cellular activities, both at the transcriptional and at the post-transcriptional level. From a physiopathological point of view, the clarification of the mechanisms governing RNA modifications may be crucial for understanding the onset and development of many diseases.^1^

Epitranscriptomics is a new area of epigenetics focusing on the aforementioned RNA modifications.^2^ Currently, more than a hundred RNA variations have been reported, including 5-methylcytosine (m^5^C), *N*^7^-methylguanosine (m^7^G), *N*^1^-methyladenosine (m^1^A), *N*^4^-acetylcitidine (Ac^4^C), pseudouridine (Ψ), 2′-*O*-methylation (N_m_), cap N^6^,2′-*O*-dimethyladenosine (m^6^A_m_), and *N*^6^-methyladenosine (m^6^A).^3,4^ The latter is the most common modification that impacts all types of RNAs across all organisms.^5−7^ The advent of DNA and RNA mapping techniques was probably the most influential in triggering the recent surge of attention toward nucleic acid modifications. Moreover, recent developments leading to single-nucleotide-resolution mapping of m^6^A have further enhanced the interest in this modification.^8^

In mammalian cells, m^6^A interferes with gene expression, thereby impacting cellular processes such as stress response and stem cell differentiation.^9^ In particular, m^6^A regulates the export, splicing, stability, and degradation of mRNAs. Thus, the presence of a methyl group on the adenosine could cause alterations in the silencing and expression of some genes.^10,11^ The m^6^A modifications are usually found in specific motifs such as DRACH (D = A/G/U, R = A/G, and H = A/C/U), RAC (R = A/G, H= A/G, and H = A/C/U), and RRACH (R = A/G, and H= A/C/U).^12−14^

Epitranscriptomic and epiprotein modifications are tightly interconnected. For instance, trimethylation on Lys36 of histone H3 (H3K36me3), a known marker for transcriptional elongation, has been indicated to guide m^6^A deposition.^15^ Specifically, the protein methyltransferase-like 14 (METTL14), which is a part of the m^6^A methyltransferase complex (MTC) along with the main m^6^A writer METTL3, recognizes and binds H3K36me3, helping the entire complex bind in a position adjacent to RNA polymerase II. In this way, the MTC encounters new RNA transcripts, leading to the cotranscriptional methylation of *N*^6^-adenosine.^15^ Chromatin immunoprecipitation sequencing (ChIP-Seq) further indicated an increase in the frequency of m^6^A patterns, which are associated with the modified histone protein, near stop codons, while m^6^A was not associated with H3K36me3 at start codons.^15^

The methylation of *N*^6^-adenosine is a complex and dynamic mechanism where several proteins play an essential role. These proteins are divided into three classes: m^6^A writers, erasers, and readers. The enzymes responsible for adenosine demethylation are the so-called erasers. The most studied eraser is the fat mass and obesity-associated protein (FTO), which is localized both in the nucleus, where it regulates RNA modifications following transcription, and in the cytoplasm, where it controls RNA metabolism.^16^ FTO catalyzes the oxidative demethylation of m^6^A and m^1^A through the formation of the hydroxyl and formyl intermediates *N*^6^-hydroxymethyladenosin (hm^6^A) and *N*^6^-formyladenosin (f^6^A), respectively.^16^ Another eraser is the alkylated DNA repair protein AlkB homologue 5 (ALKBH5), which mostly demethylates m^6^A and is present in the nucleus where it regulates RNA processing and shuttling to the cytoplasm.^17,18^ Both enzymes are dioxygenase α-ketoglutarate-dependent proteins and catalyze adenosine demethylation through a crucial Fe(II) and the cosubstrate α-ketoglutaric acid. The m^6^A readers are the members of the YT homology (YTH) domain-containing family. They recognize the methylated sites thanks to their hydrophobic pocket that is selective for the methyl group,^19^ thereby affecting changes in RNA transcription, translation, stability, and nuclear export.^19−22^ Through the recognition of m^6^A, the reader proteins contribute to the control of mRNA stability. For instance, the YTH domain-containing family proteins (YTHDF1–3) act redundantly to recognize m^6^A in the cytoplasm, mediate the degradation of mRNAs that contain the m^6^A modification, and contribute to cellular differentiation.^23,24^

**Figure 1:**
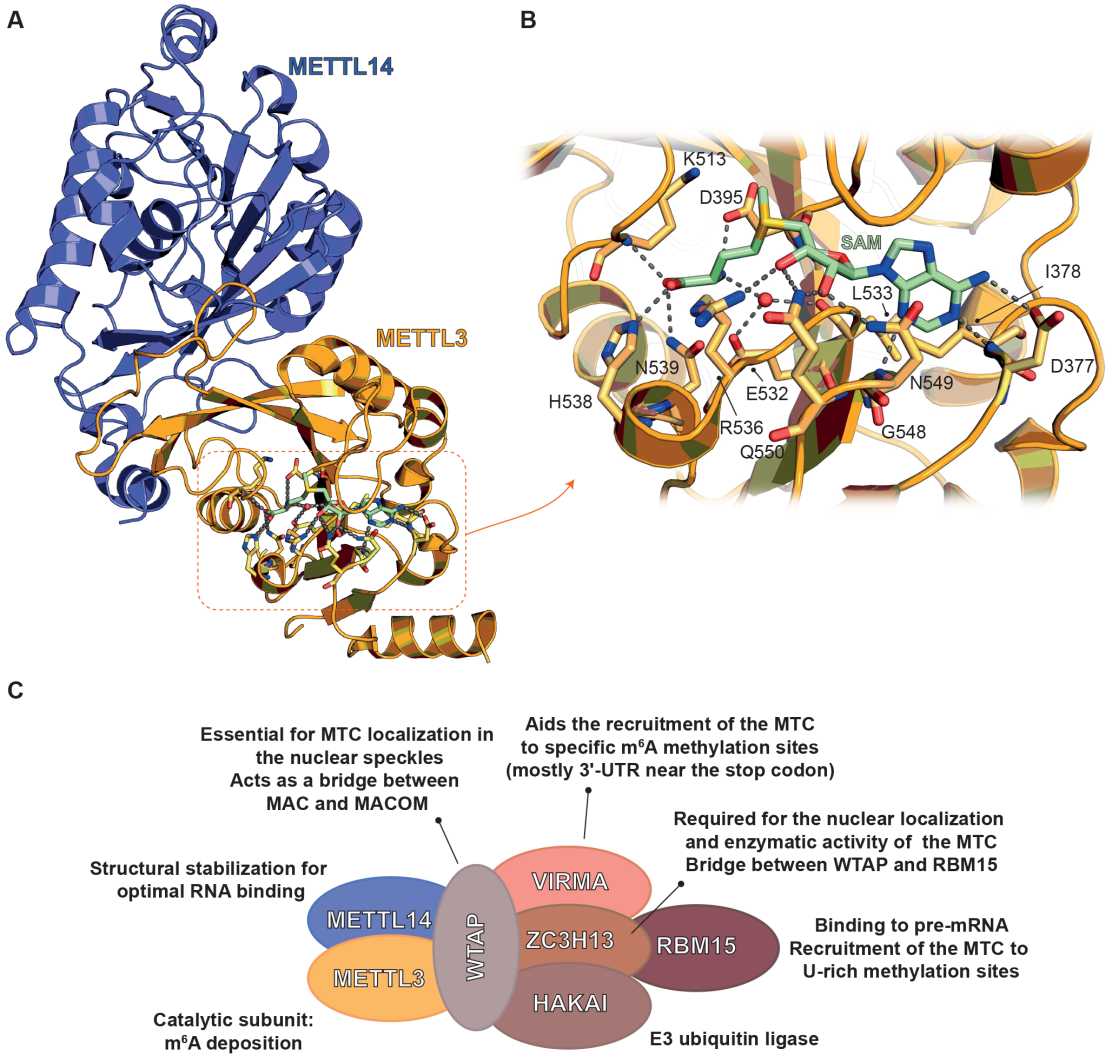
(A) Structure of METTL3–METTL14 in complex with SAM (green) (PDB ID 5IL1). (B) Focus on the catalytic pocket indicating the key interactions of SAM with METTL3 residues. Dashed gray lines indicate polar interactions, and red spheres indicate water molecules. (C) Roles and topologies of the different components of the MTC. The topology of the METTL3–METTL14–WTAP–VIRMA–ZC3H13–HAKAI complex is based on cryo-EM structures and cross-linking mass spectrometry.^26^ The connection between ZC3H13 and RBM15 is based on the literature.^50^

As anticipated above, the main m^6^A writer is METTL3, which forms a heterodimer with METTL14 (Figure 1A). In addition, the Wilms’ tumor 1-associated protein (WTAP) interacts with the dimer and is necessary to correctly deposit the m^6^A modification.^25^ The MTC also comprises other adaptor proteins such as the Vir-like m^6^A methyltransferase associated (VIRMA, also referred to as KIAA1429), the RNA-binding motif protein 15 (RBM15), the zinc finger CCCH domain-containing protein 13 (ZC3H13), and the E3 ubiquitin ligase CBLL1 (HAKAI).^26^

Although the METTL3–METTL14 complex is the main source of m^6^A in the cell, there are few other enzymes that catalyze the formation of m^6^A in a small number of RNAs.^27^ Specifically, METTL16 is able to produce the m^6^A modification in the context of the CAG sequences of the U6 small nuclear RNA (snRNA)^28^ and the mRNA of methionine adenosyltransferase 2A (MAT2A).^29,30^ In addition, the METTL5–TRMT112 complex applies the m^6^A modification to the 18S ribosomal RNA (rRNA)^31^ and ZCCHC4 targets the 28S rRNA.^32^

m^6^A modifications can influence both the structure and the folding of mRNA.^33^ Although the A–U base pairing is not compromised, the methyl rotates from a favorable (*sin*) to an unfavorable (*anti*) conformation, altering the thermodynamic stability of the double-strand.^34^ Unlike when it is present in a single strand, the methyl group acquires the *sin* conformation that probably allows an additional hydrophobic interaction with adjacent bases, stabilizing the secondary RNA structure.^35,36^ This difference can explain the different susceptibility to the degradation. Recent studies on the secondary structure of methylated RNA have shown that the adenosines near the modified base tend to assume a single-strand structure compared to those that do not possess the modification. Thus, the nuclease enzymes could find easier access and start degradation more quickly.^37^

## Structural Features of the MTC

The MTC may be divided into two subcomplexes: the m^6^A–METTL complex (MAC), which is formed by METTL3 and METTL14, and the m^6^A–METTL-associated complex (MACOM), comprising WTAP, VIRMA, ZC3H13, RBM15, and HAKAI. Thanks to the presence of a *S*-adenosyl methionine (SAM)-binding region within the methyltransferase domain (MTD), METTL3 is able to transfer a methyl group from the cosubstrate SAM to its target adenosine. Notably, although METTL14 has a MTD, it does not possess any catalytic activity because it does not have a SAM-binding pocket. Indeed, while an early report argued that both METTL3 and METTL14 possess methyltransferase activity,^38^ later crystallography studies refuted this model and suggested that the initially reported methyltransferase activity of recombinant human METTL14 expressed in insect cells was due to copurification with endogenous METTL3.^39^ Hence, METTL14 has a structural role by acting as a scaffold for the interaction between METTL3 and the RNA substrate and is essential for METTL3 catalytic activity, which is negligible in the absence of METTL14.^39,40^ Moreover, METTL14 acts as a key anchoring point for the RNA substrate through an Arg–Gly–Gly (RGG) present on its *C*-terminus.

The METTL3–METTL14 heterodimer is formed by hydrogen bonds and hydrophobic interactions occurring at MTD sites.^40^ From the crystal structure, it is evident that METTL3 interacts with METTL14 asymmetrically and in an antiparallel way. Although not all types of interactions between these proteins have been fully understood, some essential information can be deduced. The structure is butterfly shaped, with a length of 70 Å and a width of 40 Å, and the MTD of METTL3 is characterized by a Rossmann fold comprising an α–β–α sandwich, which includes eight β-sheets flanked by four α-helices and three 3_10_ helices. Through several hydrogen bonds, the SAM molecule interacts with a highly conserved Asp–Pro–Pro–Trp (DPPW) motif, and the adenine moiety is recognized by the side chain of Asp377 and the main chain of Ile378. Additionally, the methionine portion of SAM interacts with Asp395, Lys513, His538, and Asn539 directly, while a conserved water molecule bridges the contact with Glu532 and Leu533 (Figure 1B). Finally, the hydroxyl groups of the ribose establish hydrogen bonds with residues Gln550, Asn548, and Arg536.^40^ Crystallographic studies of the heterodimer also indicated that although it possesses a MTD unit, METTL14 is unfit to bind the SAM due to the lack of amino acids able to establish hydrogen bonds with the cosubstrate.^41^ Furthermore, METTL14 has a rigid structure for the presence of Trp211 and Pro362, whose side chains would clash with the adenosine portion of SAM. In analogy with the DPPW motif of METTL3, METTL14 has an EPPL motif (Glu–Pro–Pro) lacking the aromatic residues necessary for the stacking with the adenosine substrate.^41^ Notably, METTL3 also has an *N*-terminal domain that binds the WTAP factor, enabling the formation of the (WTAP–METTL3–METTL14) WMM complex.^42,43^ WTAP is the third subunit of MTC. It does not possess methyltransferase activity but facilitates the deposition of m^6^A by the MTC and is required for the localization of METTL3–METTL14 in the nuclear speckles.^42,38,44^ In mammalian cells, WTAP was shown to bind the Wilm’s tumor 1 protein, which is critical for embryonic development.^45,46^ Interestingly, WTAP levels are closely related to those of METTL3, which was shown to regulate WTAP homeostasis.^47^

Regarding the other MACOM complex subunits, VIRMA is the largest component, acting as a bridge between the different subunits. VIRMA aids the recruitment of the MTC to specific m^6^A methylation sites (roughly 60% of which are in the 3′-UTR and near the stop codon of certain mRNAs) via its interaction with WTAP, and its depletion leads to lower m^6^A levels as a consequence of limited access of the METTL3–METTL14 complex to the target mRNA.^48^ RBM15 is another adaptor protein that plays an essential role in the engagement of the MTC on the pre-mRNA.^25^ At its *N*-terminus, RBM15 has three motifs for RNA recognition, and it is involved in the recruitment of the MTC to U-rich RNA sites.^49^ ZC3H13 is a recently discovered protein that promotes the link between RBM15 and WTAP and seems to modulate the nuclear localization of the MTC.^50^ Recent cryogenic electron microscopy (cryo-EM) structures of the MACOM complex revealed that WTAP forms a homodimer that directly interacts with VIRMA to form the core of the complex and ZC3H13 stretches the conformation by binding VIRMA. Moreover, cross-linking mass spectrometry data and the cryo-EM map of the full MACOM–MAC complex uncovered the topology of the full MTC, where the interactions between METTL3 and WTAP are crucial for the formation of the complex.^26^

## Biological Roles of m^6^A

### m^6^A Implications in Noncoding RNA Functions

The m^6^A modifications are crucial in several cellular processes, including spermatogenesis, cancer progression, circadian rhythm, and viral infections.^51−55^ Notably, m^6^A is deposited not only on mRNA but also on other types of RNA consisting of noncoding sequences that influence post-transcriptional gene expression, called microRNA (miRNA). miRNAs are small sequences of 21–23 nucleotides derived from longer RNA molecules (pri-miRNA). Adenosine methylation affects miRNA biogenesis, export, and processing and the functional activity of target mRNAs. For instance, the m^6^A modification present on pri-miRNA is recognized by reader proteins (such as hnRNPA2B1, heterogeneous nuclear ribonucleoprotein A2/B1) that recruit the protein machinery, finally leading to the maturation and formation of miRNA.^56^ On the other hand, Yuan et al. reported that in mammalian cells the methylation of NOP2/Sun RNA methyltransferase 2 (NSUN2) negatively influences the biogenesis of specific miRNAs.^57^

In addition to miRNAs, the long noncoding RNAs (lncRNAs) also undergo *N*^6^-methylation. lncRNAs are essential for gene expression, since they regulate transcriptional, post-transcriptional, and translational mechanisms. For instance, in pancreatic cancer, the tumor suppressor lncRNA KCNK15 antisense RNA 1 (KCNK15-AS1) is downregulated and highly methylated, thereby destabilizing it and finally altering the expression of epithelial–mesenchymal transition (EMT) markers.^58^

### m^6^A Control of Cell Cycle and Fate

Multiple reports indicated that METTL3 activity and, consequently, the m^6^A modification are involved in the regulation of different biological processes, such as cell cycle, apoptosis, autophagy, and differentiation.

The MTC has been shown to facilitate cell cycle progression during adipogenesis by promoting cyclin A2 expression during mitotic clonal expansion.^59^ METTL3 seems to have an antiapoptotic role, and its downregulation decreases the expression of antiapoptotic regulators such as Bcl-2 while increasing the pro-apoptotic factors Bax and caspase-3 (Table 1).^60,61^ METTL3 and ALKBH5 oppositely regulate the autophagic flux by modulating the m^6^A on the transcripts of transcription factor EB (TFEB), a key regulator of lysosomal biogenesis and autophagy genes. Specifically, the METTL3-mediated addition of m^6^A on TFEB mRNA was found to reduce its translation, consequently impairing autophagy and enhancing apoptosis (Table 1).^62^

**Table 1:** Roles of METTL3–METTL14 in Biological Pathways Other than Cancer

pathway	target (function)	biological outcomes	ref
apoptosis	Bcl-2 (downregulation)	apoptosis impairment	(60, 61)
	Bax and caspase-3 (upregulation)		
apoptosis and autophagy	TFEB mRNA (methylation)	reduced TFEB translation, autophagy impairment, increased apoptosis	(62)
osteogenesis	Pth1r mRNA (methylation)	promotion of the parathyroid hormone/Pth1r signaling axis and osteogenic/adipogenic responses	(63)
osteogenesis	PI3K-AKT signaling (increase)	promotion of osteogenic differentiation	(64)
	*VEGFA*, *VEGFA-164*, and *VEGFA-188* (upregulation)		
SARS-CoV-2 infection	viral mRNA (methylation)	inhibition of RIG-I recognition and increased viral replication	(67)
atherosclerosis	FoxO1 mRNA (methylation)	higher FoxO1 expression, increased formation and migration of atherosclerotic plaques and inflammatory response	(75)
artery calcification	vascular-protecting mRNAs (methylation)	degradation of vascular-protecting transcripts and vascular calcification	(76, 77)
neural development	differentiation genes mRNAs (methylation)	promotion of neural progenitor cell differentiation	(80)
Alzheimer’s disease	AD-associated genes mRNAs (methylation)	neuroprotection	(91)

METTL3 activity seems to be essential also for osteogenic differentiation. Indeed, METTL3 deletion in mesenchymal stem cells (MSCs) was associated with impaired osteogenic potential and osteoporosis. Specifically, the parathyroid hormone/parathyroid hormone receptor 1 (Pth1r) signaling axis was shown to be a key important downstream pathway for m^6^A regulation in MSCs, and METTL knockout reduced Pth1r translation efficiency, thereby altering the parathyroid hormone-induced osteogenic and adipogenic responses (Table 1).^63^ In osteogenically differentiated bone MSCs, METTL3 is highly expressed, and its knockout impairs the phosphatidylinositol 3-kinase/AKT pathway and reduces the expression at the mRNA level of vascular endothelial growth factor A (*VEGFA*) and its splice variants *VEGFA-164* and *VEGFA-188* involved in osteogenic differentiation (Table 1).^64^

Overall, these studies show that METTL3 activity is required for cellular homeostasis maintenance, and while impairing its functions may be necessary in some conditions (such as certain cancer types), its inhibition may be potentially harmful to organism health.

### m^6^A and Viral Infections

In the viral infection context, RNA methylation can be considered from two sides, since the modification may occur on both viral and host cell RNA.^65^ For example, in the hepatitis C virus (HCV) and zika virus (ZIKV), the knockdown of host writers supports virus replication, while the knockdown of host erasers can decrease it.^65^ More recently, the COVID-19 pandemic caused by SARS-CoV-2 has prompted numerous research groups to investigate the possible correlation between this infection and epigenetic changes like m^6^A modification, which seems to have a dual activity. Typically, in viral infections, the METTL3–METTL14 complex methylates the viral RNA to distinguish it from self-RNA and finally eliminate it. Retinoic acid-induced gene I (RIG-I) is a cytoplasmic protein that acts as a sensor of viral RNA and triggers the production of interferons and many other signals for the antiviral response.^66^ Notably, during SARS-CoV-2 infection, RIG-I binds only unmethylated viral RNA. Consequently, METTL3 knockdown is critical because the related m^6^A reduction in viral RNA facilitates RIG-I binding, thereby triggering the downstream inflammatory and innate immune signaling. Moreover, METTL3 depletion was associated with a reduced expression of proviral host genes, thereby leading to a perturbation of the viral life cycle both directly and indirectly (Table 1).^67^ Similarly, silencing METTL3 or m^6^A reader YTHDF2 or YTHDF3, respectively, was associated with a significant reduction in viral replication in SARS-CoV-2 infected cells, and a similar effect (albeit to a lesser extent) was observed in cells infected with the seasonal coronavirus HCoV-OC43.^68^ Accordingly, pharmacological inhibition of METTL3 using the known METTL3 inhibitor STM2457 (compound **3b** in the METTL3 Small-Molecule Inhibitors section) impaired the replication and spread of both SARS-CoV-2 and HCoV-OC43.^68^

### m^6^A and Cardiovascular Diseases

Epitranscriptomic modifications are also critical in cardiovascular diseases. Indeed, m^6^A modifications are widely present in heart tissues and account for roughly a quarter of the total transcripts of human and mouse hearts.^69^ The maintenance of normal m^6^A levels is crucial for cardiovascular homeostasis,^70^ and multiple studies have shown a correlation between MTC dysregulation and the pathogenesis of hypertension, atherosclerosis, vascular calcification, cardiac hypertrophy, and heart failure.^71^ For instance, m^6^A levels were shown to support postnatal pulmonary hypertension in rat models.^72^ Consistently, METTL3 and METTL14 knockdown inhibits the proliferation and migration of pulmonary arterial smooth muscle cells, thereby delaying the progression of pulmonary hypertension.^73^ Similarly, in hypertension-induced hypertrophic cardiomyocytes, the amount of m^6^A-modified RNA was higher compared to that in the healthy ones.^74^

Atherosclerosis, probably the main cause of cardiovascular diseases, is also affected by the RNA m^6^A modification. Indeed, METTL14 was found to be upregulated in an endothelial cell-based TNFα-induced inflammation model. In fact, METTL14 levels are positively correlated with FoxO1 expression, which promotes the formation and migration of atherosclerotic plaques and the inflammatory response (Table 1).^75^ METTL14 was also found to be overexpressed in calcified arteries of human samples and rat models, as well as in indoxyl sulfate-treated human aortic small muscle cells (HASMCs). METTL14 overexpression is associated with higher methylation levels of vascular-protecting transcripts, thereby leading to their degradation and facilitating their calcification. Conversely, METTL14 downregulation seems to be associated with reduced calcification in HASMCs (Table 1).^76,77^ Overall, although different reports have clarified the role of m^6^A in cardiovascular pathologies, numerous mechanisms remain unclear, and further in-depth studies are needed to fully explain its role in various cardiovascular diseases.

### m^6^A in Neural Development and Disease

Although present at low levels in embryonic and postnatal brains, the amount of m^6^A modification increases significantly during adulthood.^78,79^ Accordingly, m^6^A was shown to control brain development and function in mouse models, and METTL14 knockout causes a drastic decrease of m^6^A levels in neural progenitor cells (NPCs), thus leading to numerous complications in neural development (Table 1).^80^ Indeed, in the cortex, the absence of methylation delays the maturation of neurons. In the cerebellum, m^6^A enhances mRNA degradation and alternative splicing. Conversely, METTL3 knockout provides a disruption of the granular cell layer.^79^ Moreover, in the hippocampus, the reduction of m^6^A due to METTL3 or FTO dysregulation alters neurogenesis and neuronal renewal.^79^

Fragile X syndrome is a mental pathology that manifests developmental delay and cognitive problems. The fragile X mental retardation protein (FMRP) is a polysome-associated RNA-binding protein (RBP) that hinders the translation process of some dendritic RNAs.^81−84^ The loss of this protein is related to the onset of the disease. This protein can influence the stability^85^ and export to the cytoplasm of mRNA targets,^86,87^ which are confused with methylated transcripts^79^ via direct binding. For this reason, this protein is considered a possible m^6^A reader.^88,89^ In the *Drosophila* nervous system, the presence of m^6^A limits axonal growth. Indeed, YTHDF, the only known *Drosophila* reader, interacts with Fmr1, a FMRP *Drosophila* homologue, and both can recognize the m^6^A modification, thereby regulating axonal growth. This control is critical because Fmr1 and YTHDF inhibit the expression of positive regulators of axonal growth, finally ensuring proper axonal growth and homeostasis in both the peripheral and central nervous system.^90^

In the context of neurodegenerative disorders, a recent study on Alzheimer’s disease (AD) mouse models showed reduced m^6^A methylation in AD-related genes along with slightly decreased METTL3 expression and increased FTO levels (Table 1).^91^ These studies indicate that disruption in the METTL3/FTO axis and consequent alteration of m^6^A methylation patterns influence multiple neurological pathways ranging from dendritic and synaptic development to long-term potentiation, finally strongly linking the alteration of RNA methylation to neurological pathologies.

### m^6^A and Cancer

**Figure 2:**
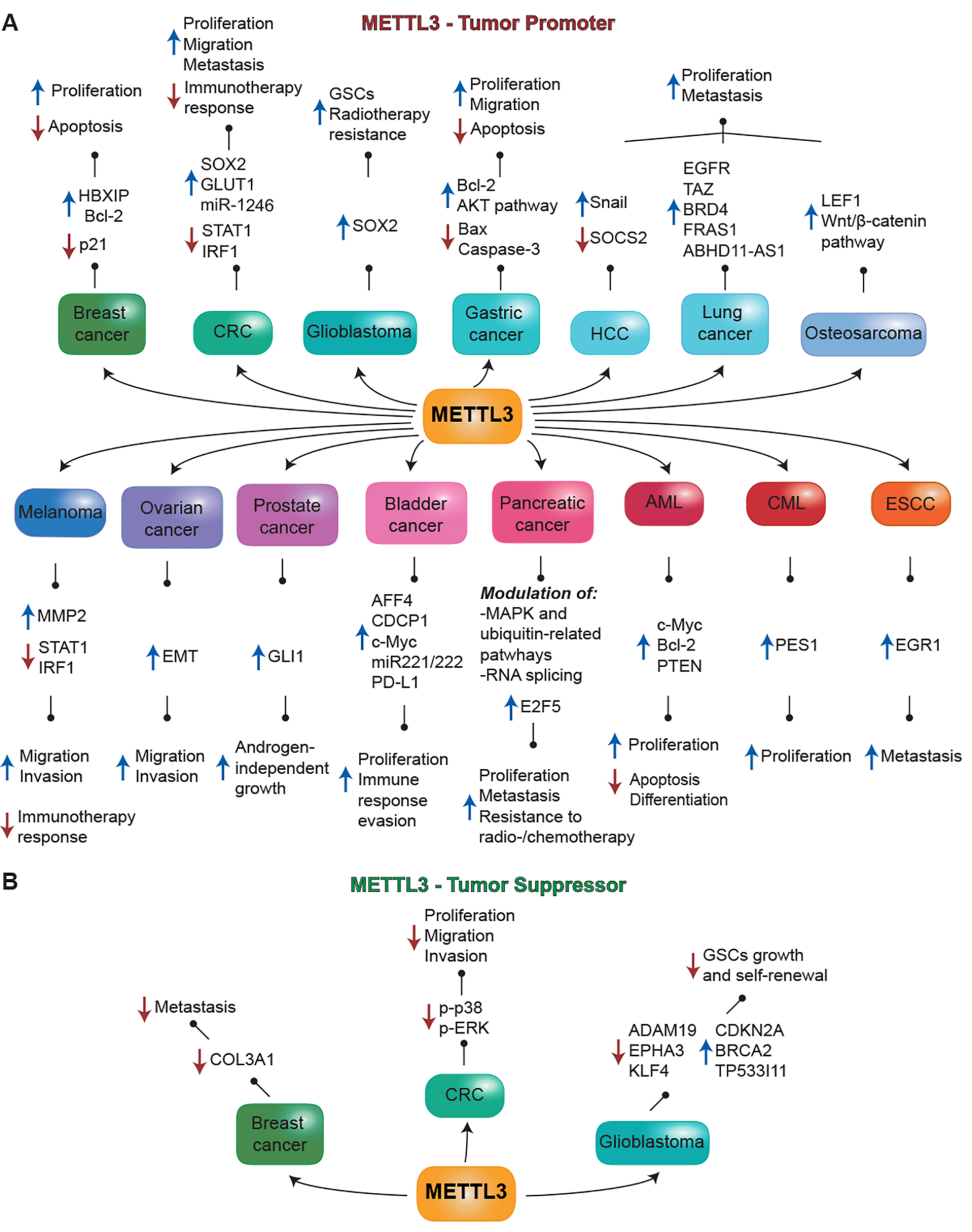
Summary of the implications of METTL3 in cancer. The figure depicts the main protein interactors and pathways regulated by METTL3 as both (A) a tumor promoter and (B) a tumor suppressor.

In human cells, the dysregulation of m^6^A impacts the normal cell cycle, thereby altering the apoptotic pathways, cell proliferation, and adhesion, which ultimately contributes to cancer development. Notably, METTL3 activity has been mostly linked to tumor-promoting functions, although in some contexts METTL3 acts as a tumor suppressor, as summarized in Tables 2 and 3 and in Figure 2. The properties of METTL3 as either a tumor promoter or suppressor have been linked to the status of p53.^92^ In response to DNA damage or oncogenic signals, METTL3 was shown to interact with and stabilize the oncosuppressor p53 and to introduce the m^6^A modification to the transcripts of several p53 target genes. In addition, *METTL3* and *p53* knockdown experiments in both lung adenocarcinoma mouse models and human cells demonstrated that METTL3 acts as a tumor suppressor specifically in the context of fully functional p53.^92^ Consequently, in circumstances where p53 activity is impaired, METTL3 may have oncogenic functions.

**Table 2:** Roles of METTL3–METTL14 as a Tumor Promoter

cancer type	target	biological outcomes	ref
breast cancer	upregulation: HBXIP, Bcl-2	increased proliferation, decreased apoptosis	(93, 94)
	downregulation: p21		
CRC	upregulation: SOX2, GLUT1, miR-1246	increased proliferation, migration, and metastasis; decreased response to immunotherapy	(97−99, 119)
	downregulation: STAT1, IRF1		
glioblastoma	upregulation: SOX2	supported the maintenance of GSCs, dedifferentiation of glioma cells, radiotherapy resistance	(101)
gastric cancer	upregulation: Bcl-2, AKT pathway	increased proliferation and migration; decreased apoptosis	(60, 103)
	downregulation: Bax, caspase-3		
HCC	upregulation: Snail	increased proliferation and metastasis	(105, 106)
	downregulation: SOCS2		
lung cancer	upregulation: EGFR, TAZ, BRD4, FRAS1, ABHD11-AS1	increased proliferation and metastasis	(107−109, 111)
osteosarcoma	upregulation: LEF1, Wnt/β-catenin pathway	increased proliferation and metastasis	(112)
melanoma	upregulation: MMP2	increased migration and invasion, decreased response to immunotherapy	(113, 119)
	downregulation: STAT1, IRF1		
ovarian cancer	upregulation: EMT	increased migration and invasion	(114)
prostate cancer	upregulation: GLI1	increased androgen-independent growth	(115)
bladder cancer	upregulation: AFF4, CDCP1, MYC, miR221/222, PD-L1	increased proliferation and evasion from immune response	(116−118)
pancreatic cancer	modulation of MAPK, ubiquin-related pathways, and RNA splicing	increased proliferation, metastasis, and resistance to radio- and chemotherapy	(121, 122)
	upregulation: E2F5		
AML	upregulation: c-Myc, Bcl-2, PTEN	increased proliferation, decreased apoptosis and differentiation	(123−125)
CML	upregulation: PES1	increased proliferation	(108)
ESCC	upregulation: EGR1	increased metastasis	(127)

**Table 3:** Roles of METTL3–METTL14 as a Tumor Suppressor

cancer type	target	biological outcomes	ref
breast cancer	downregulation: COL3A1	decreased metastasis	(96)
CRC	downregulation: p-p38, p-ERK	decreased proliferation, migration, and invasion	(100)
glioblastoma	upregulation: CDKN2A, BRCA2, TP533I11	decreased GSC growth and self-renewal	(102)
	downregulation: ADAM19, EPHA3, KLF4		

In breast cancer, the high expression of METTL3 is correlated with tumor size and aggressiveness. Moreover, METTL3 drives the aberrant expression of the oncoprotein hepatitis B X-interacting protein (HBXIP), which causes apoptosis arrest and promotes cell proliferation. Moreover, HBXIP in turn stimulates METTL3 expression by impairing the expression of let-7g, a miRNA that binds to the 3′-UTR sequence of METTL3 and downregulates its expression. Hence, a positive feedback loop connecting METTL3 and HBXIP expression is present in breast cancer, which ultimately supports tumor growth.^93^ METTL3 also decreases p21 expression, thus leading to a dysregulated cell cycle. Accordingly, the treatment with the known antidiabetic metformin led to lower levels of m^6^A on p21 transcripts, resulting in higher p21 expression and reduced tumor size in a mouse breast cancer model.^94^ Furthermore, METTL3 was shown to methylate Bcl-2 transcripts, thereby facilitating the translation of this antiapoptotic factor. Consistently, METTL3 knockdown was associated with increased apoptosis, decreased cell proliferation, and tumor growth impairment both *in vitro* and *in vivo*.^95^ Conversely, in triple-negative breast cancer (TNBC), METTL3 silencing was associated with increased metastasization. Specifically, METTL3 was able to down-regulate collagen type III α-1 chain (COL3A1), a key factor contributing to the migration, invasion, and adhesion of cancer cells.^96^

In colorectal carcinoma (CRC), METTL3 seems to have a key role by methylating the transcripts of SOX2, a transcription factor that enables the maintenance of an undifferentiated state in embryonic and pluripotent stem cells. Following m^6^A deposition, SOX2 transcripts are recognized by insulin-like growth factor 2 mRNA binding protein 2 (IGF2BP2), preventing their degradation.^97^ Moreover, METTL3 seems to promote the m^6^A–GLUT1–mTORC1 axis. Indeed, METTL3 was found to deposit the m^6^A modification on GLUT1 transcripts, thereby promoting its expression and consequently glucose uptake and lactate production. This in turn activates mTORC1 signaling, which supports CRC growth. Consistently, cancer growth was suppressed in METTL3 knockdown CRC cells and human-derived primary CRC organoids as well as METTL3 knockout mouse models.^98^ METTL3 was also found to facilitate the maturation of miR-1246 through the methylation of its pri-miRNA. miR-1246 is known to inactivate the oncosuppressor SPRED2, thus leading to its downregulation and consequent activation of the RAF/MEK/ERK pathway that supports cancer cell migration and metastasis.^99^ On the other hand, METTL3 was shown to impair proliferation, migration, and invasion in CRC cells, and its downregulation was associated with higher expression of phosphorylated p38 and ERK (p-p38 and p-ERK, respectively) and the consequent activation of the p38 and ERK signaling pathways.^100^

Like what was observed in the context of CRC, METTL3 was found to target SOX2 and consequently support the maintenance of highly tumorigenic glioma stem-like cells (GSCs) and the de-differentiation of glioma cells. Moreover, METTL3 was found to induce radiotherapy resistance through SOX2-dependent increased DNA repair, thereby acting as a key tumor promoter in glioblastoma.^101^ Conversely, Cui et al. found contrasting results, correlating a low expression of METTL3 in glioblastoma cells with a persistent stem-like state and increased GSC growth and self-renewal.^102^ Moreover, these alterations are correlated with the upregulation of oncogenic proteins such as ADAM19, EPHA3, and KLF4 and the downregulation of oncosuppressors such as CDKN2A, BRCA2, and TP533I11 in GSCs. Hence, the role of METTL3 in this type of cancer needs to be explored more deeply due to currently opposite pieces of evidence.

Recent studies demonstrate a correlation among high METTL3 levels, low FTO and ALKBH5 levels, poor prognosis, and advanced tumor stage and grade in gastric cancer.^103^ Accordingly, the downregulation of METTL3 causes an increase in pro-apoptotic protein levels, including Bax and caspase-3, and at the same time a decrease in oncogenic proteins like Bcl-2. Moreover, a decrease in the migration and proliferation of gastric cancer cells is also observed due to the inactivation of the AKT pathway as a consequence of METTL3 downregulation; this also leads to lower levels of p70S6K and cyclin D1, which usually support cell motility and replication.^60^

In hepatocellular carcinoma (HCC), METTL3 seems to have a double-faced role. Indeed, some studies suggest that a decrease in m^6^A modifications seems to promote metastasis. Indeed, the METTL3–METTL14 depletion was shown to prevent the maturation of pri-mR126 to miR126, an oncosuppressor found in low amounts in patients with metastases and relapsed forms of HCC.^104^ On the other hand, METTL3-mediated methylation leads to the degradation of the mRNA of suppressor of cytokine signaling 2 (SOCS2), an oncosuppressor whose downregulation facilitates tumor growth and metastasis.^105^ Furthermore, METTL3 activity supports HCC metastatization, as m^6^A deposition is crucial for EMT. In this context, METTL3 methylates the coding region (but not the 3′-UTR) of Snail mRNA, which in turn triggers the translation of Snail, a transcription factor that plays a pivotal role in EMT.^106^

METTL3 is also overexpressed in multiple lung cancer cell lines, where it promotes cancer cell proliferation and invasion through the deposition of the m^6^A modification onto the transcripts of EGFR and TAZ.^107^ Furthermore, through the interaction with eukaryotic translation initiation factor 3 subunit h (eIF3h), METTL3 binds to multiple mRNAs that encode for oncogenes (such as the bromodomain-containing protein 4, BRD4), promoting ribosome binding and consequent translation not through methylation but by acting as a methyl-RNA reader.^108^ Accordingly, METTL3 knockdown in A459 cells resulted in a small tumor size in mouse xenografts and higher sensitivity to the BRD4 inhibitor JQ1.^108^ In nonsmall cell lung cancer (NSCLC), METTL3 activity was found to support cell proliferation and colony formation through the methylation of mRNA encoding for Fraser extracellular matrix complex subunit 1 (FRAS1),^109^ an extracellular matrix protein that facilitates cell migration and invasion in NSCLC.^110^ Methylated FRAS1 mRNA is recognized by the m^6^A reader YTHDF1, which promotes its expression, finally leading to increased cell proliferation and invasion.^109^ The lncRNA ABHD11-AS1, identified as an oncogene in NSCLC, is methylated by METTL3, thereby increasing its stability and promoting the Warburg effect in cancer cells.^111^

High levels of m^6^A are also observed in osteosarcoma cells. Here, METTL3 activity promotes cell proliferation and invasion by regulating the mRNA levels of lymphoid enhancer-binding factor 1 (LEF1) and activating the Wnt/β-catenin pathway. Indeed, METTL3 silencing was associated with decreased m^6^A methylation and lower total levels of LEF1 mRNA and inhibited the WNT/β-catenin pathway, which is responsible for tumor progression.^112^

In melanoma, METTL3 activity augments the expression of matrix metallopeptidase 2 (MMP2), thereby increasing the motility of human melanoma cells and facilitating migration and invasion.^113^

It has been shown that METTL3 also plays a crucial role in urogenital cancers. For example, in ovarian cancer, the dysregulation of METTL3 triggers EMT, leading to the cancer cell proliferation and invasion.^114^ In prostate cancer, METTL3 is overexpressed and the methylation of the mRNA encoding for GLI family zinc finger 1 (GLI1) increases the expression of this protein, which supports androgen-independent growth. Accordingly, METTL3 knockdown stops cell growth and the invasion of prostate cancer cells.^115^ In bladder cancer, the high expression of METTL3 induces proliferation because it stimulates the transcription of AFF4, CDCP1, and c-Myc, acting as an oncogenic factor.^116^ Moreover, METTL3 activity facilitates the maturation of pri-miR221/222, which antagonizes the activity of the tumor suppressor phosphatase and tensin homologue (PTEN), thus leading to bladder cancer proliferation.^117^ Moreover, METTL3 promotes the escape of bladder cancer cells from the host immune system.^118^ Mechanistically, METTL3 methylates the 3′-UTR of the mRNA encoding for programmed death-ligand 1 (PD-L1), finally leading to higher protein expression. PD-L1 is a transmembrane protein known to contribute to the evasion of anticancer host immunity. In line with the role of m^6^A in the cancer immune response, knocking out METTL3 and METTL14 has been shown to enhance the response to immunotherapy by targeting the programmed cell death protein 1 (PD-1) in the context of melanoma and CRC resistant to immunotherapy, namely, mismatch-repair-proficient or microsatellite instability-low (pMMR-MSI-L). Mechanistically, METTL3 or METTL14 knockout decreased m^6^A at *STAT1* and *IRF1* mRNAs, thereby increasing their stability and facilitating their translation and consequent IFN-γ-STAT1-IRF1 signaling. Consequently, METTL3 or METT14 knockout tumors exhibited augmented cytotoxic tumor-infiltrating CD8+ T cells and more secretion of IFN-γ, Cxcl9, and Cxcl10.^119^

In pancreatic cancer patients, METTL3 was shown to correlate with higher stage and low survival rates, with both METTL3 mRNA and protein levels being higher in cancer cells compared to normal cells. Moreover, METTL3 knockdown in BxPC-3 and PaCa-2 pancreatic cancer cells reduced proliferation, migration, and invasion.^120^ Furthermore, METTL3 knockdown also increased sensitivity to radiotherapy and chemotherapeutics, such as 5-fluorouracil, gemcitabine, and cisplatin.^121^ cDNA microarray data combined with gene ontology and protein–protein interaction analysis suggested that METTL3’s tumor-promoting activity is correlated with its regulation of mitogen-activated protein kinase (MAPK) cascades, ubiquitin-dependent processes, and RNA splicing.^121^ Furthermore, METTL3 has been recently shown to promote pancreatic cancer growth and metastasis by enhancing the stability of the mRNA encoding the tumor promoter E2F5 through methylation.^122^

In acute myeloid leukemia (AML), METTL3 has been identified as an essential gene for cancer cell growth in two genetic screens. Accordingly, METTL3 downregulation led to cell cycle arrest and differentiation. Interestingly, METTL3 was shown to associate with chromatin independently from METTL14 and bind to specific promoters, such as those of transcription factors SP1 and SP2, where it methylates the mRNA of AML-associated genes and finally enhances their translation.^123^ Vu et al. further showed that METTL3 knockdown in human hematopoietic stem/progenitor cells (HSPCs) induces differentiation and impairs proliferation. In AML cells, where METTL3 mRNA and protein are expressed at higher levels than in HPSCs, METTL3 knockdown again led to differentiation along with apoptosis. Moreover, in MOLM-13 AML cells, METTL3-mediated mRNA methylation increases the translation of *c*-Myc, Bcl-2, and PTEN.^124^ METTL14 was also found to be overexpressed in HSPCs and in AML cells carrying t(11q23), t(15;17), or t(8;21) translocations, while its silencing induced the differentiation of both HSPCs and AML cells along with the inhibition of AML cell proliferation. Moreover, METTL14 expression was correlated with higher m^6^A deposition onto the transcripts of oncogenes *MYB* and *MYC*, which were in turn upregulated following the overexpression of METTL14.^125^

Both METTL3 and METTL14 were also found to be upregulated in different chronic myeloid leukemia (CML) cell lines and primary samples, with their silencing leading to impaired cell viability and growth. Mechanistically, METTL3 was shown to be essential for translation and ribosome biogenesis. Specifically, METTL3 methylates the mRNA encoding for the pescadillo homologue (PES1), a protein involved in the maturation of the 60S ribosomal subunit and cell cycle progression that was found to act as an oncogene in several cancers.^126^ Moreover, cytoplasmic METTL3 was proposed to act as a reader and to further support PES1 translation in a similar manner to that described in the lung cancer context.^108^

A recent study by Liao et al. indicated that METTL3 is upregulated in esophageal squamous cell carcinoma (ESCC) cells and metastatic tissues and that its activity is correlated with cancer metastasis.^127^ Cellular and *in vivo* experiments indicated that METTL3 methylates the early growth response protein 1 (EGR1) mRNA and activates the EGR1/Snail signaling, which in turn promotes metastasis. The authors also showed that the HIV drug elvitegravir promotes METTL3 degradation (see the Elvitegravir: A METTL3 Degrader section) and in turn suppresses ESCC metastasis both *in vitro* and *in vivo*.^127^

Overall, while METTL3 appears to act as a tumor suppressor in a few cases, many reports suggest that METTL3 inhibition very likely has beneficial effects in numerous cancer types (Tables 2 and 3 and Figure 2). Thus, it is of great interest for medicinal chemists to develop small-molecule inhibitors of METTL3 that may serve as both starting points for the development of new generation anticancer drugs and chemical tools to investigate the biological implications of METTL3. This new potential pharmacological approach to cancer management has been attracting increasing interest in the past few years. Hence, the purpose of the present Perspective is to provide a critical update on the state of the art of METTL3 inhibitors’ development.

## METTL3 Small-Molecule Inhibitors

### Competitive Inhibitors

Given the increasingly reported roles of METTL3 in various pathologies, it comes as no surprise that the development of METTL3 inhibitors is attracting researchers’ attention. Nonetheless, the journey of METTL3 inhibitor development started very recently; hence, only a few compounds have been reported so far. The MTD of METTL3 is regarded as the main target of inhibitor design. To this aim, initial efforts at developing METTL3 inhibitors were made by designing compounds acting as competitors of the cosubstrate SAM.

**Figure 3:**
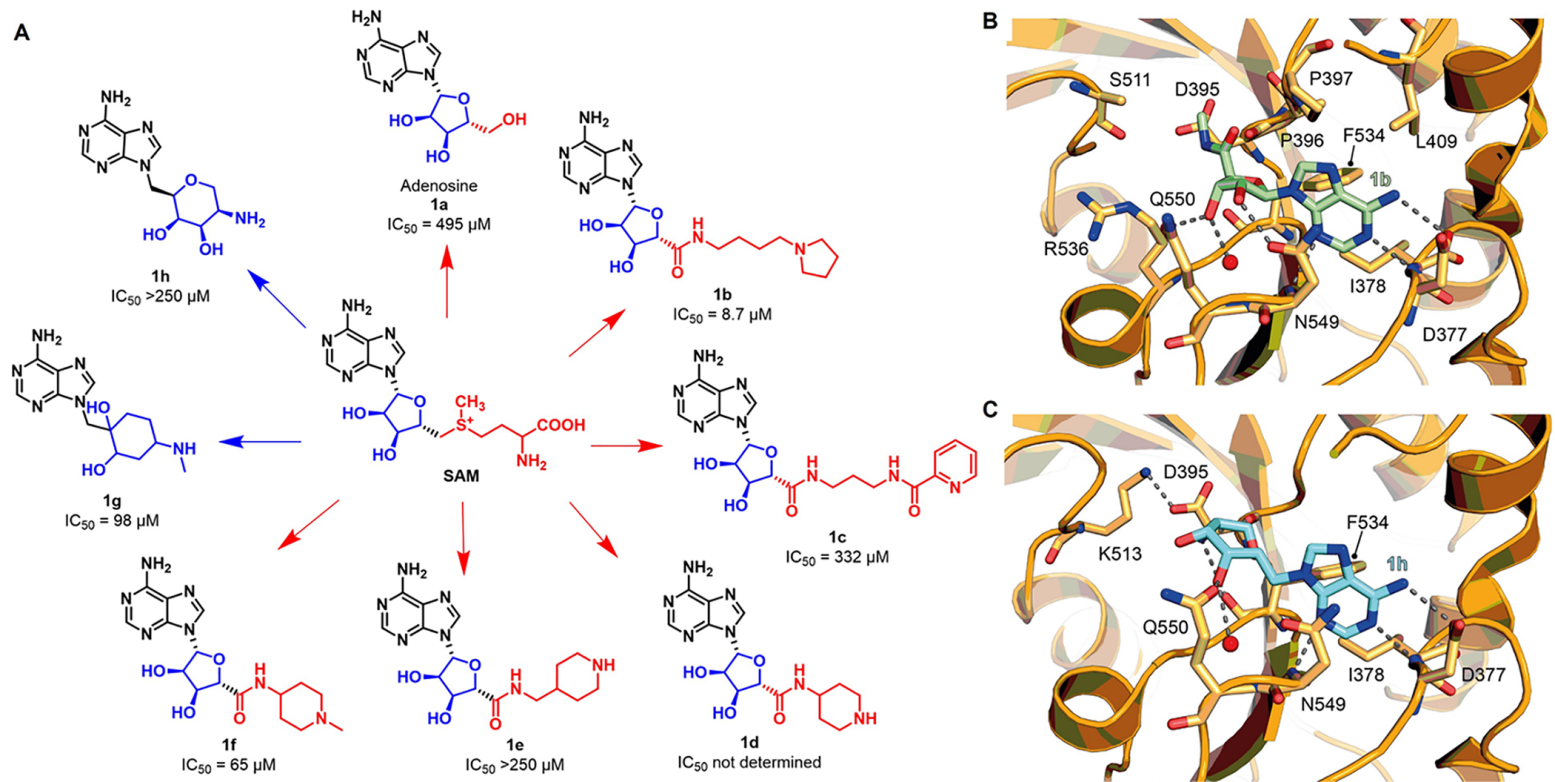
(A) Nucleoside-based METTL3 inhibitors **1a**–**h** related to cosubstrate SAM. (B) Crystal structure of METTL3–METTL14 in complex with compound **1b** (green) (PDB ID 6TTT). (C) Crystal structure of METTL3–METTL14 in complex with compound **1g** (light blue) (PDB ID 6TU1). Key residues are labeled. Dashed gray lines indicate polar interactions, and red spheres indicate water molecules.

Adenosine (**1a**) was reported as the first METTL3 inhibitor (IC_50_ = 495 μM, Figure 3A) acting with a SAM-competitive mode of action, since it overlaps with the adenosine portion of both SAM and the product SAH. Based on the adenosine scaffold, Bedi et al. performed a virtual screening on approximately 4000 compounds.^9^ They evaluated the inhibitory potency of each compound *via* a homogeneous time-resolved fluorescence (HTRF) enzyme inhibition assay developed by the same group.^128^ This assay was employed for the evaluation of all compounds developed by the Caflisch group (**1a**–**h** and **2a**–**p**). Specifically, the HTRF assay quantifies the level of m^6^A in the oligoribonucleotide substrate (50 nM) following the reaction catalyzed by METTL3–METTL14 in the presence of SAM (150 nM) and the relevant inhibitor by measuring the specific binding of the oligoribonucleotide to the m^6^A reader YTHDC1_345–509_.^128^ Among the tested molecules, 70 molecules containing the adenine and a sugar or a sugar-mimicking moiety were selected for further evaluation. Among these, only seven compounds gave promising results in the employed biochemical assays or could be cocrystallized with the METTL3–METTL14 complex. Compounds **1b**–**1f** (Figure 3A) are N-substituted amides of the ribofuranuronic acid derivatives of adenosine, while compounds **1g** and **1h** are adenosine analogues in which the ribose is replaced by a six-membered ring. Among the seven compounds, **1b** was the most potent with an IC_50_ value of 8.7 μM, over sevenfold more potent than the inhibitor **1f** (IC_50_ = 65 μM). Ribofuranuronic acid derivatives **1b**–**e** and compound **1h** could be cocrystallyzed with the METTL3–METTL14 complex and shared a conserved binding mode. The adenine portion of all compounds engages in hydrogen bonding with the backbone amide NH moieties of Ile378 and Asn549 (Figures 3B and C). The hydroxyl groups of the ribose ring of compounds **1b**–**e** form hydrogen bonds with the side chains of Asn549 and Gln550, while in compound **1h** the hydrogen bond with the Asn549 side chain is missing, although there is a primary amino group forming an ionic interaction with the Asp395 side chain along with polar interactions with the backbone carbonyl groups of both Asp395 and Phe534 (Figure 3C).^9^ In the cases of compounds **1b** and **1c**, the structure of the bound inhibitor could not be resolved beyond the amide portion, while in the compounds **1d** and **1e** it could be observed that the piperidine portion was placed in the space between the two active site loops and engaged in ionic interactions with the Asp395 and Glu481 side chains as well as van der Waals interactions with the Pro397 and Ser511 side chains. Nonetheless, repulsion with Lys513 and electrostatic desolvation of Asp395 and Lys513 decreases the binding affinity of **1d** and **1e** (IC_50_ not determined for **1d**, IC_50_ >250 μM for **1e**).^9^ Interestingly, the *N*-methylated form of **1d**, compound **1f**, exhibited a higher potency (IC_50_ = 65 μM), probably because of additional van der Waals interactions within the active site and the different p*K*_a_ of the tertiary amino group.^9^

Although compound **1b** has an IC_50_ value in the low micromolar range, it is well-known that adenosine derivatives may possess unfavorable characteristics such as low cellular permeability and poor selectivity compared to other SAM-dependent methyltransferases.^9^

**Figure 4:**
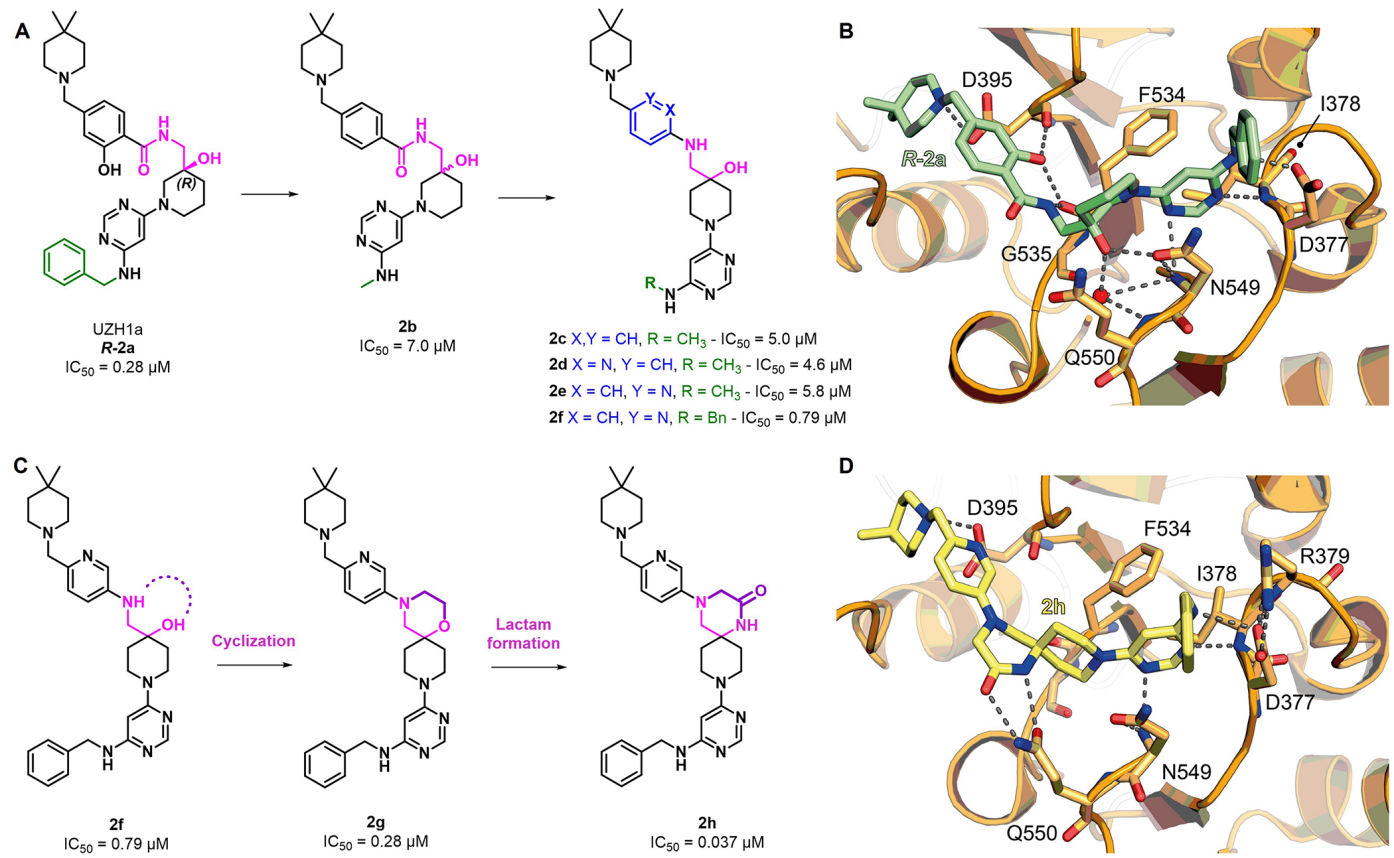
(A) METTL3 inhibitor UZH1a (***R*****-2a**) and its derivatives **2b**–**f**. (B) Crystal structure of METTL3–METTL14 in complex with compound ***R*****-2a** (green) (PDB ID 7ACD). (C) Development of compound **2h** starting from **2f**. (D) Crystal structure of METTL3–METTL14 in complex with compound **2h** (yellow) (PDB ID 7O0L). Key residues are labeled. Dashed gray lines indicate polar interactions, and red spheres indicate water molecules.

After the nucleoside-based inhibitors, the Caflisch’s team continued the research on METTL3 inhibitors with an effort to find non-nucleoside derivatives, which led to UZH1a (***R-*****2a**, Figure 4A).^129^***R-*****2a** was developed through a structure-based drug design approach (Figure 4B) and tested *via* the HTRF enzyme inhibition assay mentioned above.^128^ The authors showed that the *R*-enantiomer is 100-fold more potent than the *S*-enantiomer (UZH1b) and indicated that ***R-*****2a** is selective over a panel of other SAM-dependent methyltransferases (DOT1L, G9a, MLL4, PRDM9, PRMT1, SETD2, and SMYD3), as well as a panel of kinases. The METTL3–METTL14–***R-*****2a** cocrystal structure (Figure 4B) showed that ***R-*****2a** fits into the SAM adenosine binding pocket, with the tertiary amino group forming a salt bridge with the METTL3 Asp395 side chain. This results in the displacement of Lys513, which in turn forms a salt bridge with Glu532 that was originally formed with the amino group of SAM. These rearrangements may explain the selectivity of ***R-*****2a** over other SAM-dependent methyltransferases. In addition, hydrogen bonds are established *via* the ***R-*****2a** pyrimidine, which interacts with the backbone NH moieties of Asn549 and Ile378, and the hydroxyl group acts as a hydrogen bond donor with the side chain carbonyl of Asn549. Moreover, the pyrimidine moiety forms π-stacking interactions with the phenyl moiety of Phe534 and π–amide interactions with the side chain of Asn549. The low molecular weight and good balance between hydrophobic and hydrophilic characteristics of ***R-*****2a** justify its good cell permeability. Indeed, ***R-*****2a** decreased *N*^6^-methylation in different cell lines, such as the AML cell line MOLM-13, human bone osteosarcoma epithelial cells U2OS, and the immortalized human embryonic kidney cells HEK293T cells.^129^

Then, the Caflisch group developed further compounds possessing structures similar to that of ***R-*****2a**. These include compound **2b** (Figure 4A) bearing a methylamine moiety instead of the benzylamine present in ***R-*****2a**. Compound **2b** presented an IC_50_ of 7.0 μM in a time-resolved forster resonance energy transfer (TR-FRET) assay.^130^ Based on the conformation of **2b** in its cocrystal structure with METTL3, removing the carbonyl in the central amide along with changing the methylene connection to the piperidine from 3 to 4 seemed to enable the distance between the piperidine and phenyl ring to be maintained. Moreover, this shift led to the removal of the stereogenic center. Following this molecular simplification approach, compound **2c** was obtained, which exhibited a slight improvement in METTL3 inhibition (IC_50_ = 5.0 μM, Figure 4A) compared to **2b**. Compounds **2d** and **2e** (Figure 4A) bearing 2- and 3-pyridine in place of the phenyl ring did not show any inhibition improvement, with IC_50_ values of 4.6 and 5.8 μM, respectively. The reintroduction of the benzylamine moiety at the pyridine core in compound **2f** led to an IC_50_ value of 0.79 μM, yielding a sevenfold increase in inhibitory potency compared to **2e**, which could be attributed to an additional cation−π interaction with Arg379.^130^

**Figure 5:**
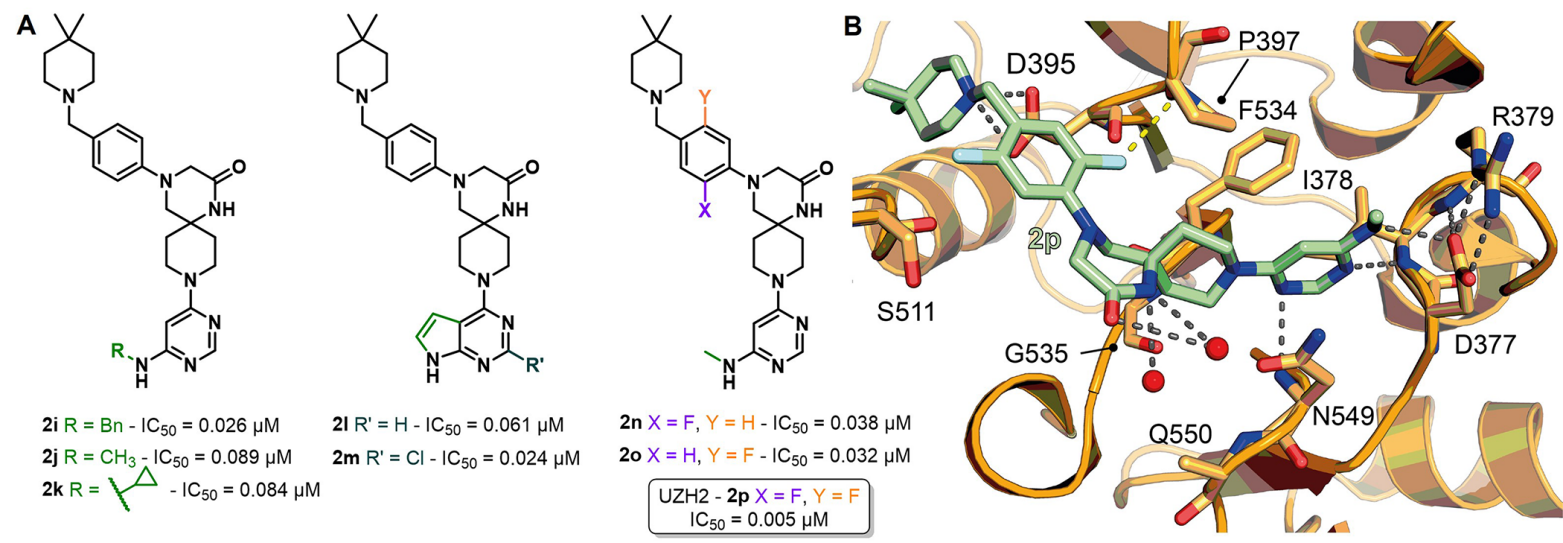
(A) Optimization strategy of the Caflisch group starting from compound **2f** derivatives bearing a phenyl ring instead of the pyridine (**2i**–**m**) and finally leading to fluorinated derivatives (**2n**–**p**), which include the single-digit nanomolar METTL3 inhibitor UZH2 (**2p**). (B) Crystal structure of METTL3–METTL14 in complex with compound **2p** (yellow) (PDB ID 7O2F). Key residues are labeled. Dashed gray lines indicate polar interactions, dashed yellow lines indicate fluorine−π interactions, and red spheres indicate water molecules.

The cyclization of compound **2f** by connecting the hydroxyl group with the neighboring aniline amine formed a spiro morpholine ring (**2g**, Figure 4C). Interestingly, **2g** exhibited an excellent inhibitory potency (IC_50_ = 0.28 μM, Figure 4C). A comparison of the binding modes of **2f** and **2g** indicated overlapping interactions except for a missing hydrogen bond between **2g** and the side chain of Gln550. Aiming to maximize the interactions between the inhibitor and METTL3, Caflisch and co-workers replaced the ether moiety of the morpholine ring with a lactam one; this led to compound **2h**, which exhibited a remarkable increase in inhibitory potency (IC_50_ = 0.037 μM, Figure 4C). Compound **2h** could form two hydrogen bonds with Gln550, one through the carbonyl group and another through the NH portion (Figure 4D). However, despite the potency improvement, **2h**, as well as **2f** and **2g**, displayed suboptimal ADME properties and poor metabolic stability. An initial approach consisted of replacing the pyridine with a phenyl ring (**2i**, IC_50_ = 0.026 μM, Figure 5A), which led to a slight increase in solubility and apparent permeability but no improvements in metabolic stability. Therefore, the replacement of the benzylamine with a methylamine resulted in compound **2j** (IC_50_ = 0.089 μM, Figure 5A), which showed augmented metabolic stability (*t*_1/2_ = 107 min, upon incubation with rat liver microsomes) and solubility at the expense of cell permeability. Interestingly, the replacement of the cyclopropyl with the methyl group on the same pyrimidine amine moiety was tolerated (**2k**, IC_50_ = 0.084 μM, Figure 5A) and led to slight improvements in solubility, cell permeability, and metabolic stability.^130^ Conversely, replacing the pyrimidine core with a pyrrolopyrimydine (**2l**, IC_50_ = 0.061 μM, Figure 5A) or 2-chloropyrrolopyrimydine (**2m**, IC_50_ = 0.024 μM, Figure 5A) while increasing the inhibitory potency was detrimental in terms of ADME properties.

Finally, the Caflisch team set out to assess the influence on the potency and ADME properties of fluorine atom(s) introduction on the phenyl ring of **2j**. This approach led to the synthesis of three compounds: the 2-fluoro derivative **2n**, the 5-fluoro derivative **2o**, and the 2,5-difluoro analogue **2p**, also indicated as UZH2. Both **2m** and **2d** exhibited slight improvements in potency, with IC_50_ values of 0.038 and 0.032 μM, respectively, and **2m** also displayed a great improvement in terms of cell permeability. Nonetheless, both compounds showed slightly lower metabolic stabilities (*t*_1/2_(**2n**) = 63 min; *t*_1/2_(**2o**) = 46 min). Remarkably, UZH2 (**2p**) was the first single-digit nanomolar METTL3 inhibitor (IC_50_ = 0.005 μM) and was highly cell-permeable, although the metabolic stability was still lower than those of its analogues (*t*_1/2_(**2p**) = 24 min). Crystallographic studies revealed that the fluorine of **2n** forms a rather unusual interaction with the nitrogen π-system of Pro397, while in **2o** it has hydrophobic contacts with Ser511 and Tyr406. These interactions, along with other polar interactions, including the salt bridge with Asp395, are kept by compound **2p**, as shown in Figure 5B. Compound **2p** was then tested in a thermal shift assays against METTL3–METTL14, METTL16, and METTL1 at both 1000 and 100 μM. At 1000 μM, it showed Δ*T*_m_ values of 3.7, 0.8, and 6.3 °C for METTL3–METTL14, METTL16, and METTL1, respectively; no Δ*T*_m_ was observed at 100 μM for METTL16 and METTL1, as compared to the shift of 4.7 °C for METTL3–METTL14. These data prove selectivity of **2p** toward other RNA methyltransferases. However, no data toward other SAM-dependent methyltransferases are available yet. Target engagement was then confirmed in HEK293T cells and AML MOLM-13 cells through the InCELL Pulse assay and CETSA, respectively. In both cases, **2p** was able to dose-dependently stabilize METTL3, with EC_50_ values of 2 and 0.85 μM, respectively.^130^ Compound **2p** also reduced the polyadenylated mRNA m^6^A/A ratio to ∼20% in MOLM-13 and prostate cancer PC-3 cells, with EC_50_ values of 0.7 μM and 2.5 μM, respectively. In addition, **2p** dose-dependently decreased MOLM-13 and PC-3 cell growth following 72 h of incubation, with GI_50_ values of 12 and 70 μM, respectively. Finally, target selectivity was also assessed in MOLM-13 cells *via* LC-MS/MS analysis of the total RNA. Results indicated no significant changes in the m^1^A/A and m^7^G/G ratios following six days of incubation at 10 μM, while small decreases were observed for m^6^A/A and m^6^A_m_/A ratios (Table 4). Overall, through this investigation, Caflisch and colleagues managed to deliver the first single-digit nanomolar METTL3 inhibitor, which may serve as a chemical probe for studying METTL3 biology and may act as a lead for further optimization.

**Table 4:**
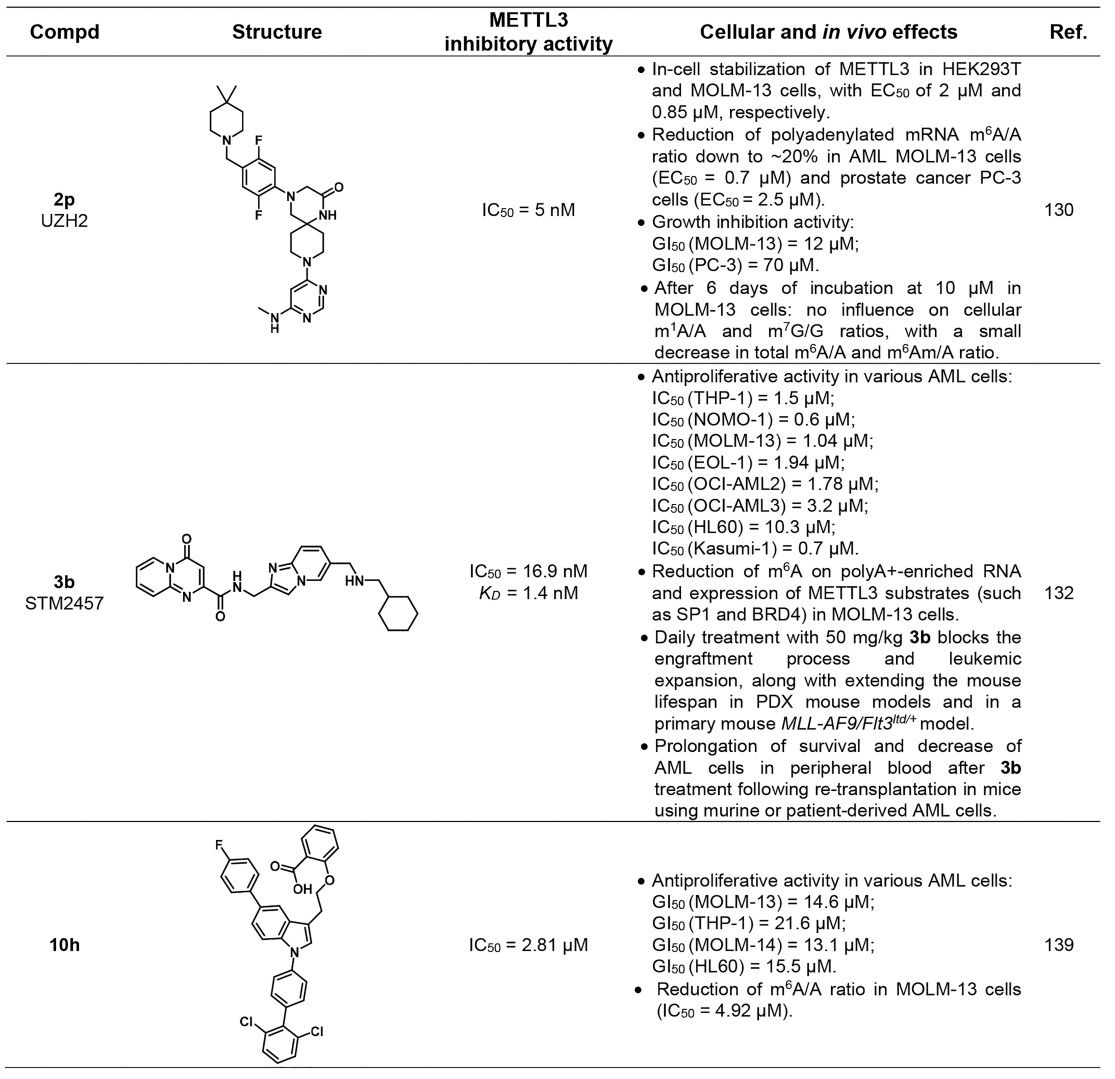
Most Relevant Competitive and Allosteric METTL3 Inhibitors

**Figure 6:**
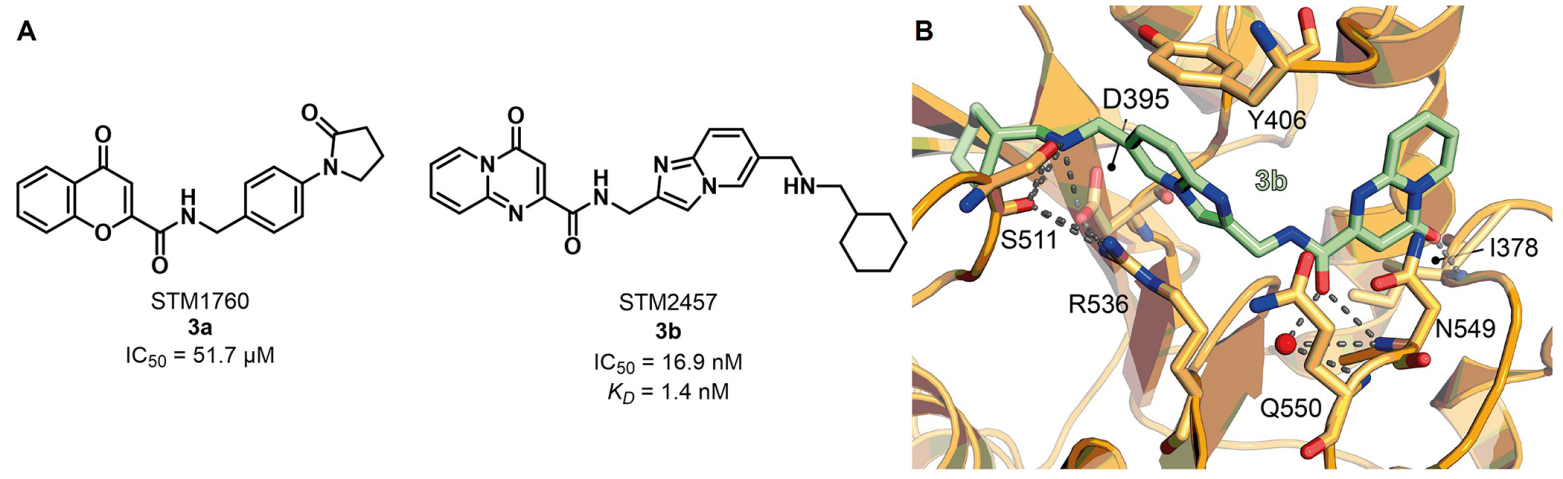
(A) Structures of the inhitial hit STM1760 (**3a**) and the optimized METTL3 inhibitor STM2457 (**3b**). (B) Crystal structure of METTL3–METTL14 in complex with compound **3b** (green) (PDB ID 7O2I). Key residues are labeled. Dashed gray lines indicate polar interactions, and red spheres indicate water molecules.

A drug discovery campaign by the Kouzarides team started with a high throughput screening (HTS) of 250 000 compounds and led to the identification of the initial hit STM1760 (**3a**, Figure 6A) possessing an IC_50_ value against METTL3 of 51.7 μM. The structural optimization of **3a** aimed at improving the *in vitro* and *in vivo* pharmacokinetics led to STM2457 (**3b**, Figure 6A), which was characterized by a pyridopyrimidone and an imidazopyridine core connected *via* an amide linker. Details of the structural optimization that culminated in **3b** are not included in the study. METTL3–METTL14 inhibitors bearing the same pyrido[1,2-*a*]pyrimidinone core linked to an indole moiety via a methylcarboxamide linker were recently reported in a patent by Storm Therapeutics (see the next subsection).^131^ Surface plasmon resonance (SPR) measurements were employed to evaluate the binding affinity and mode of action of **3b** and indicated that it acts as a SAM-competitive inhibitor with a *K*_D_ value of 1.4 nM. A RapidFire mass spectrometry methyltransferase assay using a synthetic RNA substrate (200 nM) and SAM (500 nM) as a cosubstrate indicated that **3b** has an IC_50_ value of 16.9 nM. It is worth noticing that this value may not be compared to the ones obtained for compound **2p**, as it was measured via different assays and in the presence of different concentrations of SAM. Moreover, **3b** was cocrystallized with the METTL3–METTL14 complex (Figure 6B). This structure further showed that the compound binds in a competitive manner in the SAM binding pocket, where the carbonyl function of the central amide forms two hydrogen bonds, one with Asn549 and the other one with a conserved water molecule, while the pyridopyrimidone carbonyl forms a hydrogen bond with the NH of Ile378 backbone. In addition, the secondary amine of **3b** forms a salt bridge with Asp395 and a hydrogen bond with Ser511. Compound **3b** exhibited over 1000-fold METTL3 selectivity over a panel of 45 RNA, DNA, and protein methyltransferases as well as 468 kinases. The authors hypothesize that the excellent selectivity of **3b** toward other methyltransferases comes from its structural dissimilarity and the unique binding mode when compared to SAM or other methyltransferase inhibitors known in the literature.^132^**3b** was also tested in a panel of various AML cell lines, where it impaired cell proliferation with IC_50_ values ranging from 0.7 to 10.3 μM (Table 4); meanwhile, no effects were observed for normal CD34^+^ hemopoietic cells, thus proving its lack of toxicity. Moreover, in MOLM-13 and mouse primary AML cells, treatment with **3b** induced cell cycle arrest and myeloid differentiation. **3b** also triggered apoptosis in mouse and human AML models but not in normal nonleukemic hemopoietic cells. In MOLM-13 cells, **3b** dose-dependently reduced m^6^A on poli-A^+^-enriched RNA but did not affect other RNA modifications (m^6^A_m_, m^6^_2_A, and m^7^G). To better understand how **3b** influences AML progression, Yankova et al. also studied the RNA methylation patterns in MOLM-13 cells *via* m^6^A-specific methylated RNA immunoprecipitation (m^6^A-meRIP-seq). This analysis, coupled with quantitative PCR, showed that **3b** could reduce the amount of m^6^A on polyA^+^-enriched RNA (and consequent protein expression) of METTL3 substrates, such as *SP1*, *BRD4*, and the leukemogenic factors *HOXA10* and *MYC*, while no influence on non-METTL3 mRNA substrates was observed. These pieces of evidence are essential to understand that **3b** is specific for METTL3 and that the interaction with the complex occurs in the nucleus. Subsequent studies in patient-derived xenograft (PDX) mouse models showed that daily treatment with 50 mg/kg **3b** could block the engraftment process and leukemic expansion, along with extending the mouse lifespan. Moreover, fewer human CD45^+^ cells in the spleen and bone marrow were observed along with no significant weight variations and toxicity. Moreover, treatment with **3b** led to a significant decrease in protein expression of key METTL3 m^6^A substrates, while METTL3 levels were not affected, thereby suggesting selective *in vivo* METTL3 targeting. Similar results were observed using a primary mouse *MLL-AF9/Flt3*^*Itd/+*^ model. Retransplantation experiments in rodents using murine or patient-derived AML cells from primary transplants treated with a vehicle or compound **3b** demonstrated a prolongation of survival and an evident decrease of AML cells in peripheral blood following **3b** treatment.^132^ These experiments highlight the effect of the pharmacological inhibition of METTL3 in AML, especially in preventing or prolonging the disease after transplantation. To date, **3b** is the only METTL3 inhibitor that has been fully characterized up to the *in vivo* stage, where it shows a promising therapeutic potential. It provides also the first proof of concept that the inhibition of an RNA methyltransferase by small molecules is effective in cancer.

### Patent Literature Inhibitors

The potential of METTL3 inhibition has also drawn the attention of pharmaceutical companies, with multiple compounds reported in various patents filed by Accent Therapeutics^133−135^ and Storm Therapeutics.^131,136−138^

**Figure 7:**
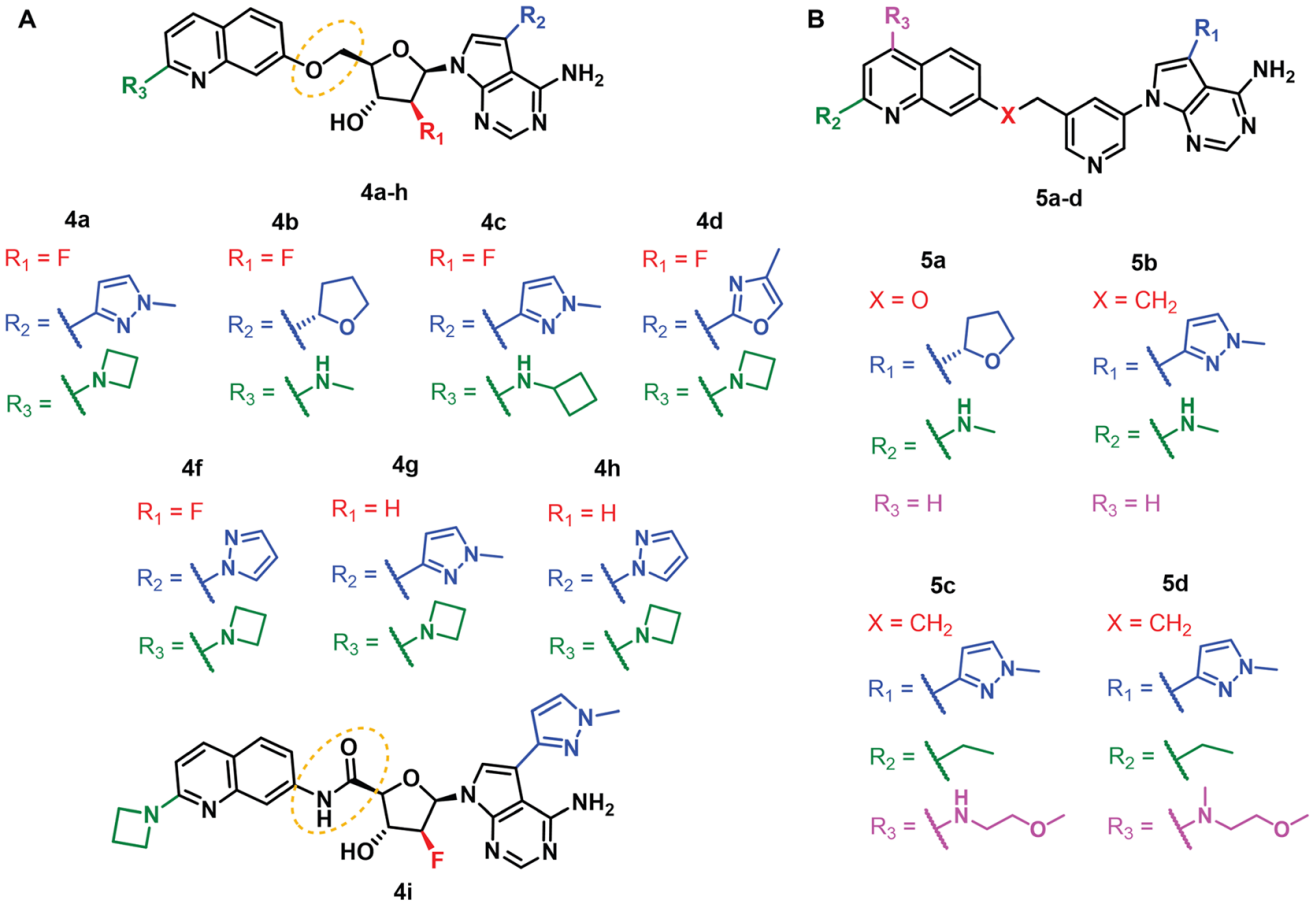
Structures of METTL3–METTL14 inhibitors (A) **4a**–**h** and (B) **5a**–**d** developed by Accent Therapeutics. All inhibitors possess IC_50_ < 10 nM.

Among the first series of compounds developed by Accent Therapeutics, a set of 2-deoxy-2-fluororibose and 2-deoxyribose derivatives bearing variously substituted 6-amino-7-deazapurine moieties at C2 and 2-aminoquinolinyl portions connected to the exocyclic hydroxyl group at C5 (compounds **4a**–**h**, Figure 7A) were the most potent and selective METTL3 inhibitors.^133^ The compounds were evaluated *via* a radiometric enzymatic assay in the presence of biotinylated RNA (100 nM) and ^3^H-SAM (100 nM). Compounds **4a**–**h** inhibited METTL3 with IC_50_ values lower than 10 nM and were >100-fold selective over PRMT5, as well as METTL1 and METTL16 in the case of **4a**. In addition, **4a**–**h** decreased the amount of m^6^A in cellular mRNA of the AML cells MOLM-13, with IC_50_ values lower than 1 μM, and impaired the proliferation of the same cells after 48 or 96 h, with IC_50_ values lower than 10 μM. Accent Therapeutics scientists also reported analogue **4i**, a derivative of **4a** bearing a carboxamide spacer at C5, which inhibited METTL3 with an IC_50_ value lower than 10 nM and >100-fold selectivity over PRMT5 and the FMS-like tyrosine kinase 3 (FLT3); however, no cellular data were provided in this case.^135^ In another patent, the central 2-deoxyribose was replaced by a pyridine core linked to the 2-aminoquinoline moiety *via* a methoxy (**5a**) or ethyl (**5b**–**d**) linker (Figure 7B). All these compounds were evaluated using the same METTL3–METTL14 radiometric assay mentioned above and exhibited potent METTL3 inhibition (IC_50_ < 10 nM) along with >100-fold selectivity over METTL1 (**5a**–**d**) and METTL16 (**5a**, **5c**, and **5d**). In addition, all compounds decreased the amount of m^6^A of total cellular MOLM-13 mRNA, with IC_50_ values lower than 1 μM, and impaired MOLM-13 proliferation after 48 (**5b**, IC_50_ < 1 μM) or 96 h (**5a**, **5c** and **5d**, IC_50_ < 10 μM).

**Figure 8:**
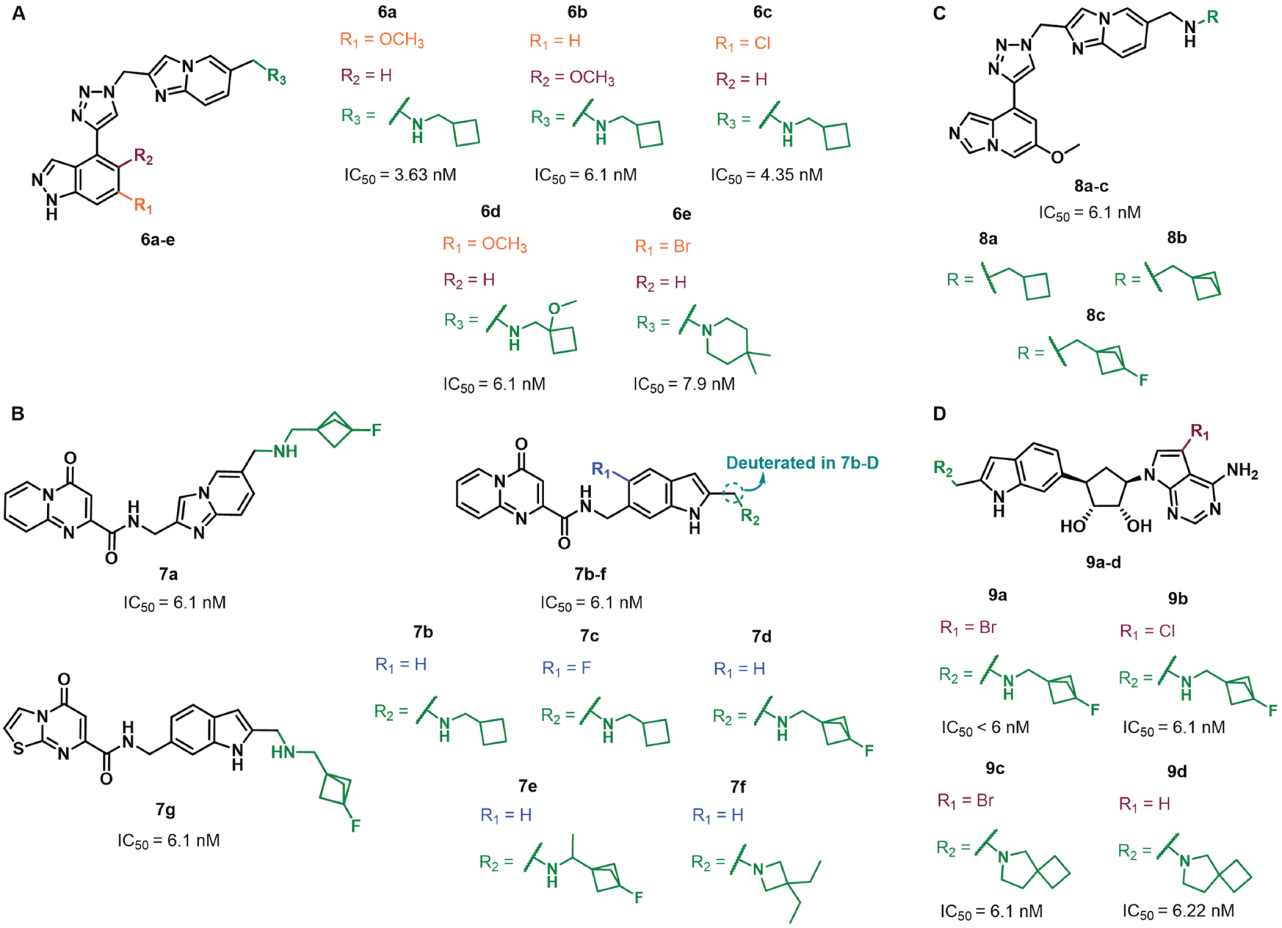
Structures of METTL3–METTL14 inhibitors (A) **6a**–**e**, (B) **7a**–**f**, (C) **8a**–**c**, and (D) **9a**–**d** developed by Storm Therapeutics. All inhibitors possess IC_50_ < 8 nM.

Storm Therapeutics scientists have also disclosed many METTL3 inhibitors possessing a diverse range of chemotypes and endowed with nanomolar potency when tested in a METTL3–METTL14 enzyme assay in the presence of 200 nM synthetic RNA substrate and 500 nM SAM. Compounds **6a**–**e**, bearing a triazole core connected to a 1*H*-indazole ring at C4 and an imidazo[1,2-*a*]pyridine moiety at N1 via a methylene linker (Figure 8A), displayed IC_50_ values of 3.63 (**6a**), 6.1 (**6b**, **6d**), 4.35 (**6c**), and 7.9 nM (**6e**).^136^ These molecules also inhibited the proliferation of the ovarian adenocarcinoma cell line Caov-3 (IC_50_ values between 182 (**6e**) and 558 nM (**6b**)) and the AML cell line MOLM-13 (IC_50_ values between 338 (**6e**) and 1.17 μM (**6b**)). In the same patent, the pyrido[1,2-*a*]pyrimidinone derivative **7a** (Figure 8B) was also reported and exhibited an IC_50_ value for METTL3 inhibition of 6.1 nM, along with the inhibition of Caov-3 and MOLM-13 cell lines with IC_50_ values of 248 and 657 nM, respectively. Analogues of **7a** were reported in a subsequent patent, which included pyrido[1,2-*a*]pyrimidinone derivatives linked to an indole moiety via a carbamoylmethyl linker (**7b**–**f**, which possess the same core as **3b**), the deuterated analogue of **7b** (**7b-D**), and the thiazolo[3,2-*a*]pyrimidinone derivative **7g** (Figure 8B).^131^ All compounds inhibited METTL3 enzymatic activity with an IC_50_ value of 6.1 nM and impaired the proliferation of the Caov-3 cells (IC_50_ values between 80 (**7d**) and 237 nM (**7b**)) and the AML cell line Kasumi-1 (IC_50_ values between 263 (**7d**) and 587 nM (**7b**)). Similar results were obtained for compounds **8a**–**c** (Figure 8C), all of which exhibited an IC_50_ value for METTL3 inhibition of 6.1 nM.^137^ Compounds **8a**–**c** inhibited Caov-3 cell proliferation, with IC_50_ values of 103, 110, and 305 nM, respectively, and displayed IC_50_ values against Kasumi-1 cell proliferation of 316 nM, 419 nM, and 1.15 μM, respectively.

Compounds **9a**–**d**, bearing a cyclopentane-1,2-diol core linked to a 6-amino-7-deazapurine at C3 and an indol-6-yl moiety at C5 (Figure 8D), displayed single-digit IC_50_ values for METTL3 inhibition (IC_50_ (**9a**) < 6 nM, IC_50_ (**9b**) = 6.1 nM, IC_50_ (**9c**) = 6.1 nM, and IC_50_ (**9d**) = 6.22 nM).^138^ Similar to other compound series from Storm Therapeutics, these compounds were tested for their influence on the ovarian cancer Caov-3 and AML Kasumi-1 cell proliferation, with IC_50_ values in the 250–556 nM range for Caov-3 and those in the 0.95–1.39 μM range for Kasumi-1. It is worth noticing that, differently from Accent Therapeutics, the Storm Therapeutics patents did not report any data regarding target selectivity.

### Allosteric Inhibitors

**Figure 9:**
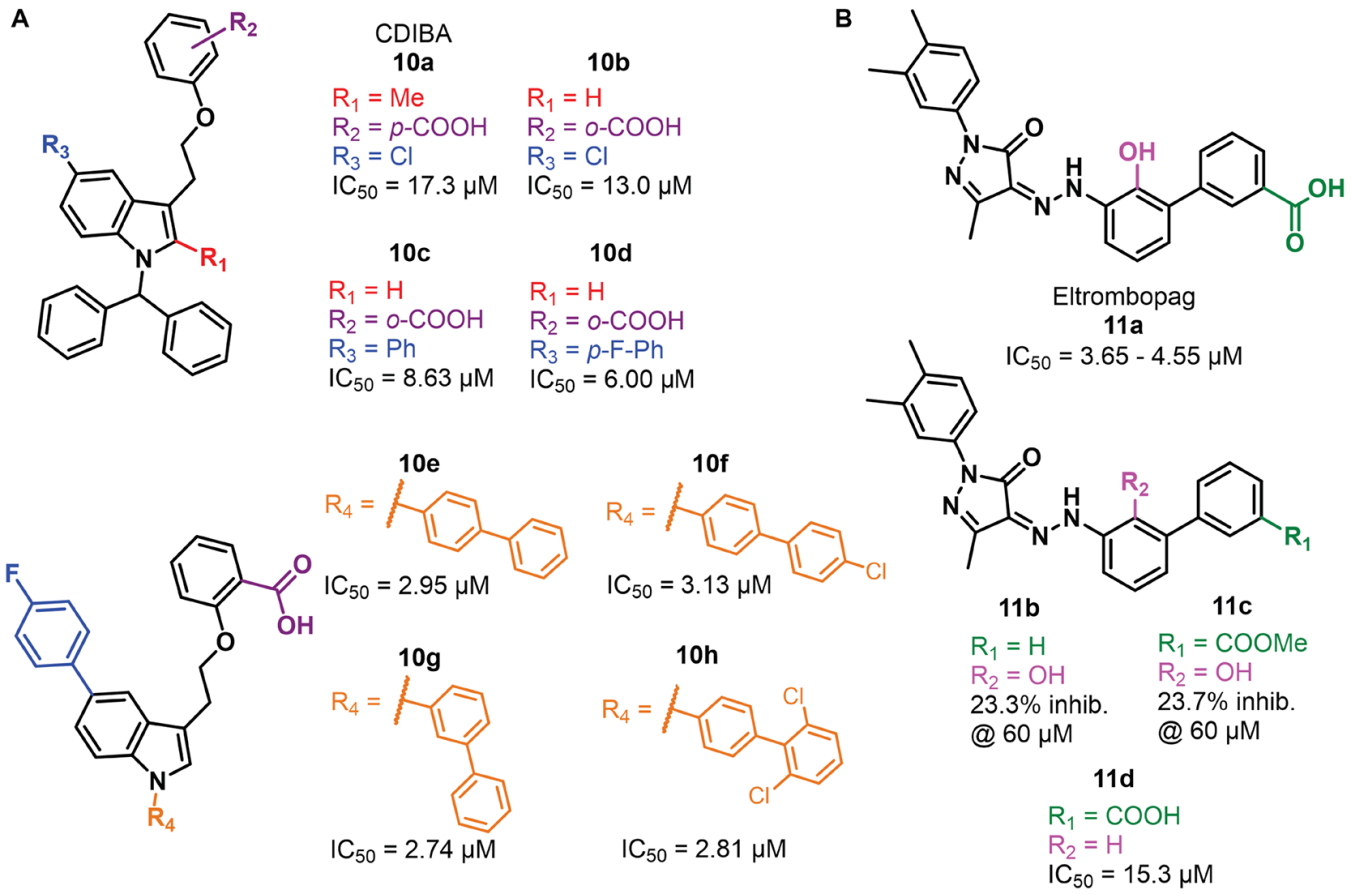
(A) Structures of METTL3 allosteric inhibitors **10a**–**h**. (B) Structures of eltrombopag (**11a**) and its inactive analogues **11b**–**d**.

Another possible approach for reducing METTL3 enzymatic activity involves the use of allosteric inhibitors characterized by a reversible and noncompetitive interaction with the METTL3–METTL14 complex. The first METTL3 allosteric inhibitor reported in the literature is **10a** (CDIBA), a 4-[2-[5-chloro-1-(diphenylmethyl)-2-methyl-1*H*-indol-3-yl]-ethoxy] benzoic acid (Figure 7A) identified through a screening of a Korea Chemical Bank compound library that displayed an IC_50_ value toward METTL3–METTL14 of 17.3 μM.^139^ Notably, **10a** was previously reported in the literature as a cytosolic phospholipase A_2_ (cPLA_2_) inhibitor.^140,141^ An optimization study performed on **10a** aimed at improving the METTL3 inhibitory activity indicated that the removal of the methyl group at C2 of the indole ring is tolerated and shifting the carboxy group of the benzoic acid moiety from the *para* to *meta* position is beneficial for the inhibitory activity. Because the methyl at C2 was demonstrated to be important for cPLA_2_ inhibition,^139^ the authors opted to carry on the optimization process from compound **10b**, which lacks the methyl at C2, beyond having the carboxyl moiety in the *meta* position. Replacing the chlorine atom at C5 of the indole core of **10b** with a phenyl ring, as in **10c**, also increased the inhibitory potency (IC_50_ = 8.63 μM). The latter was further improved by adding an electron-withdrawing substituent on the phenyl ring in the *para*-position, with fluorine giving the best results, as indicated by the IC_50_ value of **10d** of 6.0 μM (Figure 7A). Finally, replacing the diphenylmethyl moiety of **10d** with differently substituted and/or oriented biphenyl moieties led to a twofold rise in potency in the case of compounds **10e**–**h**, exhibiting IC_50_ values of 2.95, 3.13, 2.74, and 2.81 μM, respectively (Figure 9A). These four compounds were tested in AML MOLM-13 cells and were able to dose-dependently decrease cell proliferation, with EC_50_ values of 32.5, 26.9, 29.9, and 14.6 μM, respectively. The most potent compound **10h** was also shown to impair cell proliferation of AML cell lines THP-1, MOLM-14, and HL60, with GI_50_ values in the 13–22 μM range (Table 4). Moreover, it was able to suppress m^6^A/A ratio levels in MOLM-13 cells. Finally, the IC_50_ value of **10h** was shown not to be influenced by SAM or RNA substrate concentration, thereby indicating that it possesses an allosteric mode of action, although the specific allosteric site remains still unknown.

Recently, eltrombopag (**11a**, Figure 9B), a known agonist of the thrombopoietin receptor used for the treatment of chronic immune thrombocytopenia and aplastic anemia, has been also proposed as a potential allosteric inhibitor of the METTL3–METTL14 complex.^142−144^ Compound **11a** was assayed *via* bioluminescence and mass spectrometry-based assays and showed IC_50_ values of 3.65 μM and 4.55 μM, respectively, while SPR analysis indicated a *K*_D_ value of 13.2 μM. Selectivity profiling indicated no influence on the activity of five histone methyltransferases (DOT1L, G9a, PRMT1, SETD2, and SMYD3) at a concentration of 10 μM, with a slight influence on the MLL4 complex (29% inhibition at 10 μM) and PRDM9 (30% increase in activity at 10 μM). Like **10h**, the IC_50_ value of **11a** was not affected by different SAM or RNA substrate concentrations, suggesting an allosteric mode of action. According to docking studies, **11a** binds to an allosteric binding site distinct from the SAM pocket, with the carboxylic acid moiety forming hydrogen bonds with the backbone amides of Asp499 and Cys550, while the phenol forms a hydrogen bond with the carboxylate group of Asp453 and the hydrazine moiety forms a hydrogen bond with the carboxamide group of Gln496. Moreover, **11a** forms extensive van der Waals interactions with aromatic residues and hydrophobic amino acids such as Val452, Val485, and Val487.^145^ The importance of the carboxylic acid for the METTL3–**11a** interaction was confirmed by the massive drop in inhibitory potency caused by the removal or esterification of the carboxylic acid (compound **11b** or **11c**, respectively). Similarly, the removal of the phenolic hydroxyl (**11d**) group caused a fourfold decrease in potency (Figure 7B).^145^ Compound **11a** was then tested in a cellular context, where it inhibited the AML MOLM-13 cell line growth (GI_50_ = 8.28 μM), and dose-dependently reduced the m^6^A levels after 24 h of treatment. Moreover, **11a** displayed synergistic antiproliferative activity when tested in MOLM-13 cells in combination with the AML approved drug venetoclax, known BCL-2 inhibitor, while weak or no synergy was observed in combination with other AML drugs such as gliterinib, cytarabine, and sorafenib.^146^

### Natural Products

**Figure 10:**
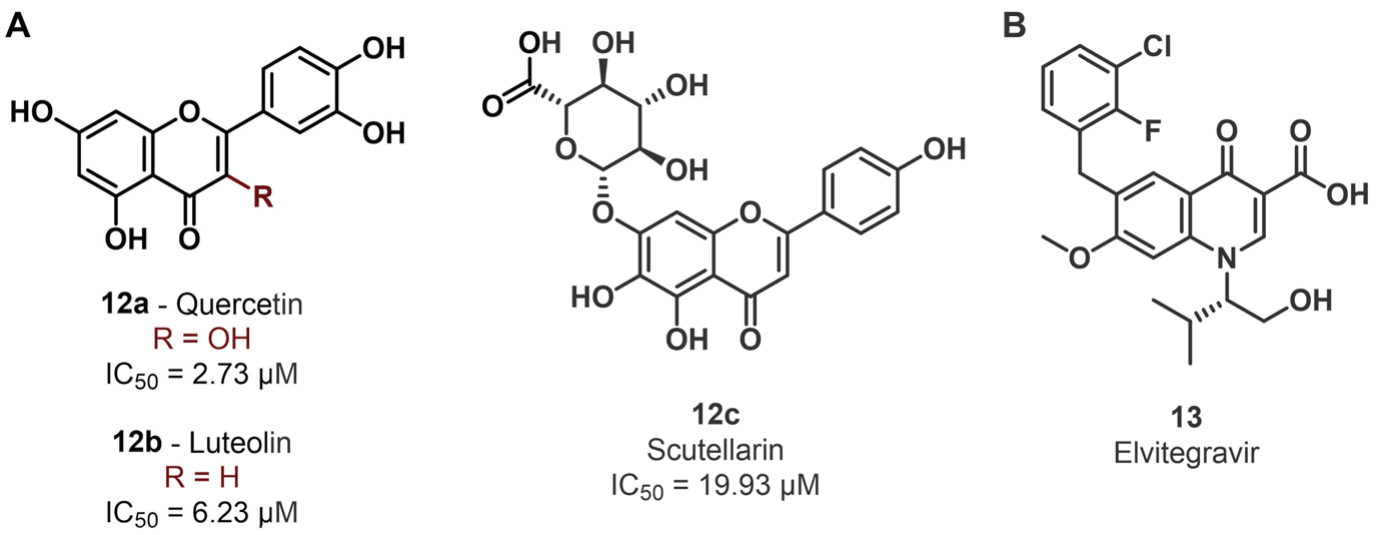
(A) Structures of natural products **12a**–**c** reported as METTL3 inhibitors. (B) Structure of the METTL3 degrader elvitegravir (**13**).

Recently, a virtual screening carried out on natural products identified the flavonoids quercetin (**12a**), luteolin (**12b**), and scutellarin (**12c**) as METTL3 inhibitors (Figure 10A). *In vitro* evaluation confirmed their METTL3 inhibition potential, with IC_50_ values of 2.73, 6.23, and 19.93 μM, respectively. **12a** was then tested in the human pancreatic adenocarcinoma cell line MIA PaCa-2, where it could decrease the m^6^A/A ratio only at concentrations of 200 and 400 μM and impaired cell viability at micromolar concentrations (IC_50_ = 73.5 μM).^147^ Similarly, an IC_50_ value of 99.97 μM was observed in the pancreatic cancer cell line Huh7. Molecular docking revealed several hydrogen bonds between the flavone core’s hydroxyl groups and Arg536, Asn549, Cys376, and Ile378, as well as hydrophobic interactions between the flavone C6′ group and Phe534 and Pro397 in addition to π–π interactions between Phe534 and the pyrone moiety. From these studies, it seems that **12a** is able to fill the SAM adenosine binding pocket but not the methionine one.^147^ Although this suggests that **12a** may act as a competitive inhibitor, the reported study is missing any binding mode analysis. Indeed, it lacks the determination of the IC_50_ values in the presence of SAM or of the substrate. Consequently, the mode of action of this compound still needs to be fully clarified.

Moreover, it is crucial to highlight that data obtained with polyphenolic compounds should be taken with care because they are known to have pleiotropic activity (*e.g*., **12b** has also been indicated to modulate multiple epigenetic enzymes, such as DNMT1, HDAC1, p300, SIRT6, but also topoisomerases I and II)^148^ and may also interfere with biochemical assays.^149^ Hence, these molecules should not be used as chemical probes to study METTL3–METTL14 activity but rather represent starting points for the development of new optimized derivatives.

### Elvitegravir: A METTL3 Degrader

Finally, it is worth mentioning that the integrase inhibitor elvitegravir (**13**, Figure 10B), currently marketed as an anti-HIV treatment, has been shown to interact with METTL3.^127^ Liao et al. measured a *K*_D_ value of 4.79 nM *via* SPR experiments, although no fitting was provided for *K*_D_ calculation and the lowest tested concentration in the binding experiments was 3.125 μM. The authors also showed that **13** could decrease METTL3 protein levels and inhibited the invasion capability of the ESCC cell lines KYSE270 and KYSE150-Luc-LM5 at 5 and 10 μM with no effects on cell proliferation. Experiments performed on mice intravenously injected with KYSE150-Luc-LM5 cells a showed dose-dependent reduction of lung metastasis in the group treated with **13** (at either 5 or 10 mg/kg). Mechanistically, **13** was shown to promote METTL3 degradation by facilitating its interaction with the ubiquitin E3 ligase STIP1 homology and U-Box containing protein 1 (STUB1), as confirmed by both Western blot and functional experiments performed on ESCC cells.^127^

## Conclusions

Although m^6^A modifications have been known for about 50 years, we have seen an increasing interest in epitranscriptomic research in the past few years.^150^ Indeed, especially in the last five years, numerous studies have shed light on the physiological and pathological functions of RNA modifications. Several diseases are associated with aberrant m^6^A methylation patterns, and epitranscriptomics has evolved from a niche topic to an active and rapidly evolving research field. However, many efforts need still to be made to understand the complex interplay of dynamic and reversible RNA modifications by readers, writers, and erasers. In a multidisciplinary approach, medicinal chemists may aid to shed light on the underlying complex biology.

The RNA writer METTL3–METTL14 affects the RNA metabolism directly or indirectly, thus impacting its downstream processing. As a result of aberrant METTL3–METTL14-mediated RNA methylation, irregular cellular processes, as outlined above, might have severe implications for the onset and/or progression of various diseases such as viral infections, cardiovascular pathologies, neurologic disorders, and cancer. As outlined above, METTL3 inhibition may be beneficial for understanding its implications in cellular homeostasis and pathology and might aid the development of innovative drugs. Accordingly, METTL3 inhibitors have started to appear in the literature in the recent years.

Because METTL3 is a SAM-dependent methyltransferase, it is not surprising that the earliest prototypes of METTL3 inhibitors were SAM structural analogs.^129^ The Caflisch team conducted two subsequent medicinal chemistry campaigns strongly supported by crystallography studies to obtain more potent inhibitors. The first study led to compound UZH1a (***R*****-2a**),^129^ which exhibited selective submicromolar METTL3 inhibition along with good inhibitory potency but unfavorable ADME properties. The second study consisted of a structure-based drug discovery campaign aimed at improving the potency and the ADME properties of this compound series. This approach led to the reduction of ***R*****-2a** structure’s flexibility through the introduction of spiro bicyclic rings, as in compounds **2g, 2h, 2i**, and **2j**. The latter showed nanomolar METTL3 inhibition but still no favorable ADME properties. To improve the latter compounds, Caflisch and co-workers performed a series of modifications, among which the introduction of two fluorine atoms on the benzene core led to UZH2 (**2p**) possessing a single-digit nanomolar IC_50_ value along with acceptable ADME properties.^130^ Furthermore, **2p** is selective over two other RNA methyltransferases, and its cellular selectivity over other RNA methyltransferases was confirmed in the AML cell line MOLM-13 via LC-MS/MS experiments.

The most advanced inhibitor identified so far is STM2457 (**3b**), which was recently reported by Yankova et al. Compound **3b** is a highly selective and potent METTL3 inhibitor with a good pharmacokinetics, exhibiting promising anticancer activity both *in vitro* and *in vivo*. In more detail, **3b** displayed cancer-selective micromolar to submicromolar antiproliferative activity values in a panel of various AML cell lines, along with increased lifespan, no significant weight variations, and no toxicity in AML PDX mouse models.^132^ These experiments highlight the efficacy of the pharmacological inhibition of METTL3 in AML.

Beyond **3b**, numerous nanomolar METTL3 inhibitors have been reported in patents filed by Accent Therapeutics (**4a**–**h** and **5a**–**d**) and Storm Therapeutics (**6a**–**e**, **7a**–**g**, **8a**–**c**, and **9a**–**d**). All of them exhibited antiproliferative activity in the low micromolar to submicromolar range in different cancer cell lines, including AML (both Accent and Storm compounds) and ovarian cancer (Storm compounds only).

Finally, allosteric inhibitors have been recently shown to be valuable alternatives to METTL3 catalytic inhibitors, although research efforts are still necessary to find potent and selective compounds of this type. Nonetheless, initial results obtained with CDIBA (**10a**) and its derivative **10h**, as well as eltrombopag (**11a**), have demonstrated that METTL3–METTL14 possesses allosteric pockets that may be exploited for its modulation in future studies. In addition, given the essential role of YTHDF proteins in mediating the effects of the m^6^A modification, inhibitors of these reader proteins may have similar effects as METTL3 inhibitors. To this end, a recent study indicated that the organoselenium drug ebselen covalently binds and inhibits YTHDF proteins and sets the ground for the development of a new class of m^6^A inhibitors.^151^

We are still in the early stages of the journey toward potent and selective METTL3 inhibitors, and many biological questions regarding the mechanism and function of the METTL3–METTL14 complex remain unanswered. Indeed, the implications of METTL3 activity are controversial in some contexts such as breast cancer, CRC, and glioblastoma, where it seems to have a dual role, and METTL3 has crucial physiological roles in cell cycle regulation, differentiation, and neural development. Furthermore, the inhibition of the m^6^A erasers FTO and ALKBH5 has been shown to be beneficial in cancers such as glioblastoma, breast cancer, pancreatic cancer, and AML. Remarkably, in two cases, both METTL3 and FTO or ALBKBH5 inhibitors resulted effective anticancer agents even in the same AML cell line.^152−154^ Consequently, the disruption of METTL3–METTL14 activity may potentially contribute to the impairment of essential physiological processes, thereby leading to detrimental outcomes. Therefore, a multidisciplinary approach is needed to further clarify METTL3–METTL14 biology in the more general context of epitranscriptomics in order to fully validate it as a manageable drug target.

One way to elucidate the activity of METTL3 would rely on chemical knockdown approaches, for instance, through the development of specific proteolysis targeting chimeras (PROTACs).^155^ The perspective highlights the currently known compounds that can inhibit METTL3 and may be employed as chemical probes, as well as possible lead compounds for future research programs. The available cocrystal structures of METTL3–METTL14 bound to its inhibitors will aid the rational design of new molecular entities. To this aim, solving the structures of METTL3–METTL14 bound to allosteric inhibitors will open new avenues for the development of new tools and potential drugs. The exciting journey has just begun, and we will soon see a surge in epitranscriptomics research focusing not only on biological aspects but also on potential therapeutic avenues.

## Abbreviations Used

ADAM19: a disintegrin and metalloprotease domain 19
AFF4: ALF transcription elongation factor 4
AKT: Ak strain transforming
ALKBH5: alkylated DNA repair protein alkB homologue 5
AML: acute myeloid leukemia
Bax: Bcl-2-associated X protein
Bcl-2: B-cell lymphoma-2
BRCA2: breast cancer gene 2
BRD4: bromodomain-containing protein 4
BxPC-3: human primary pancreatic adenocarcinoma cell line
CDCP1: CUB domain-containing protein-1
CDIBA: 4-[2-[5-chloro-1-(diphenylmethyl)-2-methyl-1*H*-indol-3-yl]-ethoxy] benzoic acid
CDKN2A: cycline dependent kinase inhibitor 2A
ChIP-Seq: chromatin immunoprecipitation sequencing
CML: chronic myeloid leukemia
cPLA_2_: cytosolic phospholipase A_2_
COL3A1: collagen-type III α-1 chain
CRC: colorectal carcinoma
cryo-EM: cryogenic electron microscopy
DOT1L: disruptor of telomeric silencing 1-like
EGR1: early growth response protein 1
eIF3h: eukaryotic translation initiation factor 3 subunit H
EMT: epithelial-mesenchymal transition
EPHA3: ephrin type-A receptor 3
ERK: extracellular signal-regulated kinase
ESCC: esophageal squamous cell carcinoma
f^6^A: *N*^6^-formyladenosine
FLT3: FMS-like tyrosine kinase 3
FMRP: fragile X mental retardation protein
FoxO1: forkhead box protein O1
FRAS1: Fraser extracellular matrix complex subunit 1
FTO: fat mass and obesity-associated protein
GLI1: GLI family zinc finger 1
GLUT1: glucose transporter 1
GSC: glioma stem-like cells
HASMCs: human aortic small muscle cells
HBXIP: hepatitis B X-interacting protein
HCC: hepatocellular carcinoma
hm^6^A: *N*^6^-hydroxymethyladenosine
hnRNPA2B1: heterogeneous nuclear ribonucleoprotein A2/B1
HSPCs: hematopoietic stem/progenitor cells
HTRF: homogeneous time-resolved fluorescence
IGF2BP2: insulin-like growth factor 2 mRNA binding protein 2
KCNK15-AS1: KCNK15 antisense RNA 1
KLF4: Kruppel-like factor 4
LEF1: lymphoid enhancer-binding factor 1
lncRNA: long noncoding RNAs
m^6^A: *N*^6^-methyladenosine
MAC: m^6^A-METTL complex
MACOM: m^6^A-METTL-associated complex
MAPK: mitogen-activated protein kinase
MAT2A: methionine adenosyltransferase 2A
METTL: methyltransferase-like protein
miRNA: microRNA
MMP2: matrix metallopeptidase 2
MSCs: mesenchymal stem cells
MTC: methyltransferase complex
MTD: methyltransferase domain
mTORC1: mammalian target of rapamycin complex 1
NOP: nucleolar protein 2
NPCs: neural progenitor cells
NSUN2: NOP2/Sun RNA Methyltransferase 2
p70S6K: 70 kDa ribosomal protein S6 kinase
PaCa-2: pancreatic cancer cell line
PD-L1: programmed death-ligand 1
PDX: patient-derived xenograft
PES1: pescadillo homologue
PRDM9: PR domain zinc finger protein 9
PRMT: protein arginine methyltransferase
PTEN: phosphatase and tensin homologue
Pth1r: parathyroid hormone receptor 1
RAF: rapidly accelerated fibrosarcoma
RBM15: RNA-binding motif protein 15
RBP: RNA binding protein
RIG-I: retinoic acid-induced gene I
rRNA: ribosomal RNA
SAM: *S*-(5′-adenosyl)-l-methionine
SARS-CoV-2: severe acute respiratory syndrome coronavirus 2
SETD2: SET domain containing 2
SMYD3: SET and MYND domain containing 3
Snail: zinc-finger transcription factors
snRNA: small nuclear RNA
SOCS2: suppressor of cytokine signaling 2
SOX2: SRY-Box transcription Factor 2
SPR: surface plasmon resonance
SPRED2: sprouty related EVH1 domain containing 2
SUN: Sad1p UNC-84
TNBC: triple-negative breast cancer
STUB1: STIP1 homology and U-Box containing protein 1
TFEB: transcription factor EB
TR-FRET: time-resolved Forster resonance energy transfer
U2OS: human bone osteosarcoma epithelial cells
UTR: untraslated region
VEGFA: vascular endothelial growth factor A
VIRMA: vir-like m6A methyltransferase associated
WMM: WTAP–METTL3–METTL14 complex
WNT: wingless-related integration site
WTAP: Wilms’ tumor 1 associating protein
YAP: yes1 associated transcriptional regulator
YTH: YT homology
YTHDF: YTH *N*^6^-methyladenosine RNA binding protein
ZC3H13: zinc finger CCCH domain-containing protein 13
ZIKV: Zika virus
